# DunedinPACNI estimates the longitudinal Pace of Aging from a single brain image to track health and disease

**DOI:** 10.1038/s43587-025-00897-z

**Published:** 2025-07-01

**Authors:** Ethan T. Whitman, Maxwell L. Elliott, Annchen R. Knodt, Wickliffe C. Abraham, Tim J. Anderson, Nicholas J. Cutfield, Sean Hogan, David Ireland, Tracy R. Melzer, Sandhya Ramrakha, Karen Sugden, Reremoana Theodore, Benjamin S. Williams, Avshalom Caspi, Terrie E. Moffitt, Ahmad R. Hariri

**Affiliations:** 1 Department of Psychology and Neuroscience, Duke University, Durham, NC USA; 2 Department of Psychology, Center for Brain Science, Harvard University, Cambridge, MA USA; 3 Department of Psychology, University of Otago, Dunedin, New Zealand; 4 Department of Medicine, University of Otago, Christchurch, New Zealand; 5 New Zealand Brain Research Institute, Christchurch, New Zealand; 6 Department of Neurology, Christchurch Hospital, Waitaha Canterbury, Te Whatu Ora–Health New Zealand, Christchurch, New Zealand; 7 Department of Medicine, University of Otago, Dunedin, New Zealand; 8 Dunedin Multidisciplinary Health and Development Research Unit, Department of Psychology, University of Otago, Dunedin, New Zealand; 9 Te Kura Mahi ā-Hirikapo, School of Psychology, Speech and Hearing, University of Canterbury, Christchurch, New Zealand; 10 Pacific Radiology Canterbury, Christchurch, New Zealand; 11 King’s College London, Social, Genetic, and Developmental Psychiatry Centre, Institute of Psychiatry, Psychology, & Neuroscience, London, UK; 12 PROMENTA, Department of Psychology, University of Oslo, Oslo, Norway; 13 Department of Psychiatry and Behavioral Sciences, Duke University, Durham, NC USA

**Keywords:** Predictive markers, Neural ageing

## Abstract

To understand how aging affects functional decline and increases disease risk, it is necessary to develop measures of how fast a person is aging. Using data from the Dunedin Study, we introduce an accurate and reliable measure for the rate of longitudinal aging derived from cross-sectional brain magnetic resonance imaging, that is, the Dunedin Pace of Aging Calculated from NeuroImaging (DunedinPACNI). Exporting this measure to the Alzheimer’s Disease Neuroimaging Initiative, UK Biobank and BrainLat datasets revealed that faster DunedinPACNI predicted cognitive impairment, accelerated brain atrophy and conversion to diagnosed dementia. Faster DunedinPACNI also predicted physical frailty, poor health, future chronic diseases and mortality in older adults. When compared to brain age gap, DunedinPACNI was similarly or more strongly related to clinical outcomes. DunedinPACNI is a next-generation brain magnetic resonance imaging biomarker that can help researchers explore aging effects on health outcomes and evaluate the effectiveness of antiaging strategies.

## Abstract (web summary)

Differences in the Pace of Aging are important for many health outcomes but difficult to measure. Here the authors describe the Dunedin Pace of Aging Calculated from NeuroImaging measure, an approach that uses a single brain image to measure how fast a person is aging and can help predict mortality or the risk of developing chronic disease.

## Main

Aging is the gradual, progressive and correlated decline of multiple organ systems over decades. Longitudinal studies provide evidence for substantial individual variation in the rate of aging; people born in the same year can age slower or faster than their peers^1–3^. Furthermore, aging itself is increasingly regarded as a potentially preventable cause of chronic disease. Accordingly, accurate and reliable measures of how fast a person is aging are needed to effectively study how individual variation in the rate of aging contributes to disease risk and to evaluate interventions intended to slow aging before irreversible decline^4–8^.

Age-sensitive alterations in DNA methylation, referred to as epigenetic clocks, are currently the most widely used measures for estimating individual differences in aging^4,9,10^. First-generation epigenetic clocks were trained on chronological age^11,12^, but the more precisely they predicted chronological age, the less well they predicted clinical outcomes^13,14^. In response, second-generation clocks were trained on measures of health that predict mortality in older people^15–17^. However, these clocks were trained on cross-sectional phenotypes in multiage samples, not on longitudinal observations of the same person as recommended in geroscience^5,18^. This limitation led to the development of a third-generation longitudinal approach to measuring aging.

We previously adopted this longitudinal approach in the Dunedin Study, which has followed a population representative sample of 1,037 people born in the same year (1972–1973) from birth to age 45 (ref. ^19^). Across two decades (ages = 26, 32, 38 and 45 years), we repeatedly measured 19 biomarkers of cardiovascular, metabolic, renal, immune, dental and pulmonary functioning. By averaging the decline in the trajectories of these biomarkers, we operationalized the theoretical construct of biological aging into a specific measure that we called the Pace of Aging^2^. We subsequently developed an epigenetic clock that accurately and reliably estimates the Pace of Aging: the Dunedin Pace of Aging Calculated from the Epigenome (DunedinPACE)^20^. Because DunedinPACE is calculated from a single time point measurement of DNA methylation, it has been rapidly adopted by studies on aging, where it has been associated with signs of accelerated brain aging, morbidity and mortality^20–25^. However, it has not been possible to export DunedinPACE or other epigenetic clocks to studies lacking DNA methylation data. This includes many neuroimaging studies of brain aging and neurodegenerative diseases such as Alzheimer’s disease (AD).

Current neuroimaging-based approaches to measure aging, akin to first-generation epigenetic clocks, involve training models to predict chronological age from variability in magnetic resonance imaging (MRI) measures of brain structure in large multiage samples^26–30^. Researchers then typically quantify a brain age gap, which reflects the difference between a participant’s predicted and actual chronological age. A positive brain age gap is interpreted as evidence of accelerated brain aging. As with first-generation epigenetic clocks, these age deviation approaches unavoidably mix model error (for example, historical differences in environmental exposures, survivor bias, disease effects, measurement bias) with a person’s true rate of biological aging^31–33^.

**Fig. 1:**
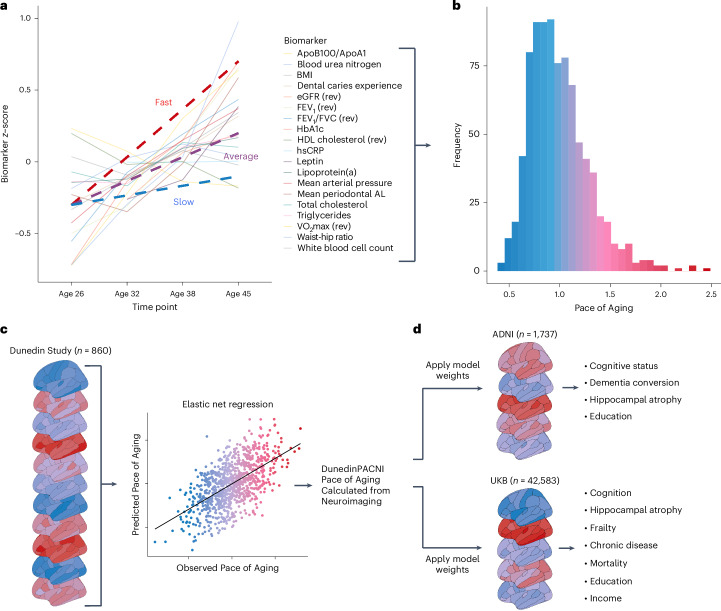
Schematic overview of the study methods. **a**, Plot of mean scores for all 19 biomarkers comprising the Pace of Aging across four waves of observation at ages 26, 32, 38 and 45 years in the Dunedin Study. Hypothetical individual trajectories are shown for people with relatively slow, average and fast Pace of Aging from ages 26 to 45 years. **b**, Distribution of Pace of Aging scores in Dunedin Study members at age 45. Warmer colors represent a faster Pace of Aging; cooler colors represent a slower Pace of Aging. **c**, A single T1-weighted MRI scan collected from 860 Dunedin Study members at age 45 years was used to train an elastic net regression model to predict the Pace of Aging. We call the resulting measure DunedinPACNI. **d**, Regression weights from the DunedinPACNI model developed in the Dunedin Study were applied to T1-weighted MRI scans collected in the ADNI and UKB datasets to derive DunedinPACNI scores. Those scores were then related to aging-related phenotypes. AL, attachment loss; Apo, apolipoprotein; BMI, body mass index; FEV_1_, forced expiratory volume in 1 s; eGFR, estimated glomerular filtration rate; HbA1c, glycated hemoglobin; HDL, high-density lipoprotein; hsCRP, high sensitivity C-reactive protein; VO_2_max, maximal oxygen uptake. Source data

In this study, using a single T1-weighted MRI scan collected at age 45 in the Dunedin Study, we describe the development and validation of a brain MRI measure for the Pace of Aging (Fig. 1a–c). We call this measure Dunedin Pace of Aging Calculated from NeuroImaging (DunedinPACNI). Using data from the Human Connectome Project (HCP), we evaluated the test–retest reliability of DunedinPACNI. Exporting the measure to the Alzheimer’s Disease Neuroimaging Initiative (ADNI), UK Biobank (UKB) and Latin American Brain Health Institute (BrainLat) datasets, we conducted a series of preregistered analyses (https://rb.gy/b9x4u6) designed to evaluate the utility of DunedinPACNI for predicting multiple aging-related health outcomes (Fig. 1d). To benchmark our findings, we compared the effect sizes for DunedinPACNI to those for brain age gap^34^. DunedinPACNI is a brain-based measure trained to directly estimate longitudinal aging of non-brain organ systems. Therefore, if DunedinPACNI indeed estimates individual differences in the rate of aging, it would add evidence for close links between brain integrity and whole-body aging and establish neuroimaging as a powerful tool for measuring aging, that is, not just of the brain but of the entire body^35^.

## Results

### DunedinPACNI: a brain MRI measure of longitudinal aging

**Extended Data Fig. 1:**
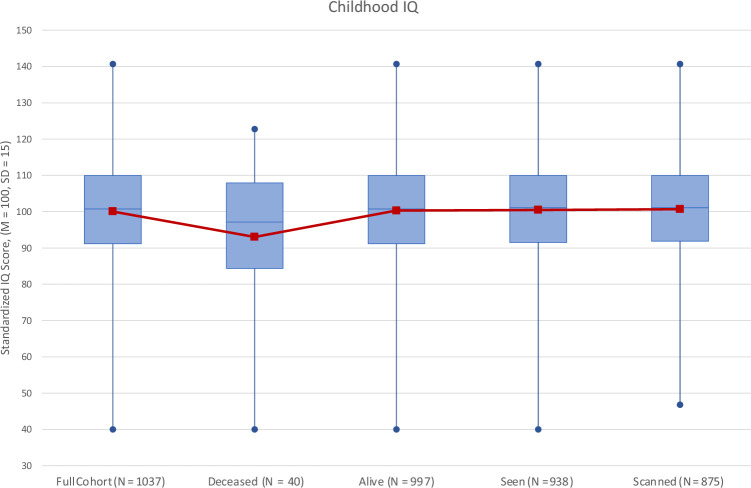
Dunedin Study attrition analysis using IQ. No significant differences in childhood IQ were found between the full cohort, those still alive, those seen at Phase 45, or those scanned at Phase 45. Those who were deceased by the Phase 45 data collection had significantly lower childhood IQ’s than those who were still alive (t = 2.09, p = 0.04). Center lines of boxes represent the median. Lower and upper hinges of boxes represent the 25th and 75th percentiles. Whiskers show the range. The red line connects the means of each distribution. We report childhood IQ because it is known to be a strong predictor of late-life health outcomes, as shown by many cohort studies from many nations. Childhood IQ predicts health and social outcomes in adulthood, and these outcomes include physical functions, cognitive decline, mental health, inflammation, metabolic syndrome, disease incidence, dementia, mortality, and also neuroimaging-based, genomic, and epigenetic indicators of health. Based on the literature, we report three groups: study members who died before age 45, and thus could not have taken part in data collection, study members who were alive and thus could take part, and study members who actually did take part. We compared these three groups to the original birth cohort. The figure shows that the small group of study members who had died before age 45 had significantly lower mean childhood IQ as a group. Some of the early deaths were Dunedin Study members who had more disadvantages in their lives leading to poorer health and increased risk of early mortality. Study members who died of childhood diseases may have been already unwell at the time of IQ testing, which could have lowered their scores. However, cohort members who are still alive and cohort members who took part in data collection did not differ from the full original cohort on their mean childhood IQ; they still represent population variation on this key health risk factor. Abbreviations: IQ = intelligence quotient. Source data

**Extended Data Fig. 2:**
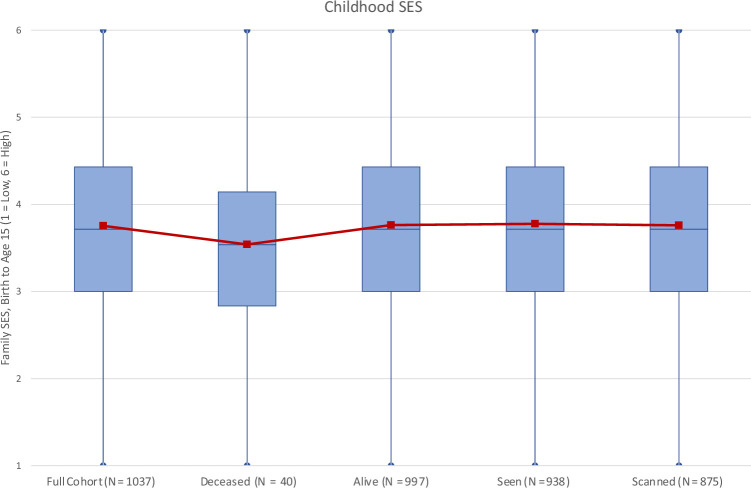
Dunedin Study attrition analysis using SES. No significant differences were found between the full cohort, those deceased, those alive, those seen at Phase 45, or those scanned at Phase 45 on childhood SES. Center lines of boxes represent the median. Lower and upper hinges of boxes represent the 25th and 75th percentiles. Whiskers show the range. The red line connects the means of each distribution. We report childhood SES because it is known to be a strong predictor of late-life health outcomes, as shown by many cohort studies from many nations. Childhood SES separately predicts health and social outcomes in adulthood, and these outcomes include physical functions, cognitive decline, mental health, inflammation, metabolic syndrome, disease incidence, dementia, mortality, and also neuroimaging-based, genomic, and epigenetic indicators of health. Based on the literature, we report three groups: study members who died before age 45, and thus could not have taken part in data collection, study members who were alive and thus could take part, and study members who actually did take part. We compared these three groups to the original birth cohort. The figure shows that the small group of study members who had died before age 45 had somewhat lower mean childhood SES as a group. Some of the early deaths were Dunedin Study members who had more disadvantages in their lives leading to poorer health and increased risk of early mortality. However, cohort members who are still alive and cohort members who took part in data collection did not differ from the full original cohort on their mean childhood SES; they still represent population variation on this key health risk factor. Abbreviations: SES = socioeconomic status. Source data

We trained an elastic net regression model to predict the longitudinal Pace of Aging measure using T1-weighted MRI scans collected in a subsample of 860 Dunedin Study members when they were 45 years old. This subsample maintains the population representativeness of the full cohort (Extended Data Figs. 1 and 2). Specifically, the elastic net regression model used 315 MRI-derived structural measures for each study member, including regional cortical thickness (CT), surface area (SA), gray matter volume (GMV) and gray:white signal intensity ratio (GWR), as well as subcortical gray matter and ventricular volumes^36^. We performed tenfold cross-validation to identify the optimal tuning parameters^37^. This optimized model was used to create DunedinPACNI.

**Fig. 2:**
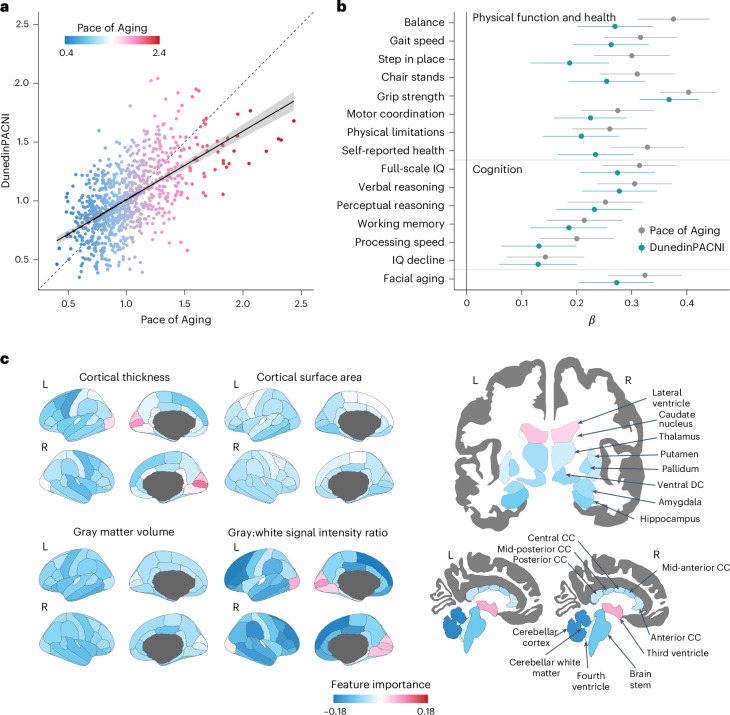
DunedinPACNI model validation and feature importance. **a**, In-sample correlation between Pace of Aging and DunedinPACNI. Warmer colors represent a faster Pace of Aging and cooler colors represent a slower Pace of Aging. The regression error band represents the 95% CI. **b**, Comparison of absolute effect sizes for associations between DunedinPACNI and Pace of Aging with physical functioning, cognition and subjective aging measures in 860 members of the Dunedin Study. Effects are presented as standardized *β* coefficients with the error bars as the 95% CIs. **c**, Covariance between MRI-derived brain features and Pace of Aging. Of the 315 brain features used in model training, 216 were set equal to zero because of the high correlation between brain measures and to reduce overfitting. The 99 features included in the final model are visualized in Supplementary Fig. 1. Warmer colors represent features that positively covaried with DunedinPACNI scores (that is, a larger value indicates faster aging), while cooler colors represent features that negatively covaried with DunedinPACNI scores (that is, a larger value indicates slower aging). Features that did not contribute to the estimation of DunedinPACNI predictions are shown in gray. CC, corpus callosum; DC, diencephalon; IQ, intelligence quotient; L, left; R, right. Source data

The in-sample correlation between DunedinPACNI and the longitudinal Pace of Aging was *r* = 0.60 (Fig. 2a). We performed a cross-validation analysis by splitting the sample into training and testing subsets 100 different times. Each time, we used 90% of the sample for training and held out the remaining 10% for testing. Across all 100 different splits, the average correlation between DunedinPACNI and Pace of Aging in the testing sample was *r* = 0.42. This prediction accuracy is in line with next-generation epigenetic biomarkers of aging^15,20,38^. For both DunedinPACNI and the longitudinal Pace of Aging, higher scores indicate faster aging. Note that DunedinPACNI is a quantitative variable that indexes relative differences between people without clear units. For this reason, we restricted our interpretation to directional comparisons within studies. Associations between faster DunedinPACNI scores and measures of physical functioning, cognitive functioning and facial aging were similar to those previously observed with Pace of Aging^2^. In these analyses, we controlled for sex but not age because all Dunedin Study members have the same chronological age. The DunedinPACNI effect sizes for 12 of the 15 measures were within the 95% confidence intervals (CIs) of the original Pace of Aging (Fig. 2b; full results in Supplementary Table 1). This was expected given the high internal correlation between DunedinPACNI and Pace of Aging. Dunedin Study members with faster DunedinPACNI scores had worse balance, slower gait, weaker lower-body and upper-body strength, and poorer coordination; they also reported worse health and more physical limitations; performed more poorly on tests of cognitive functioning; experienced greater childhood-to-adulthood cognitive decline; and looked older. These results indicate that DunedinPACNI accurately estimates the longitudinal Pace of Aging in the Dunedin Study dataset.

### DunedinPACNI reflects canonical patterns of brain aging

The optimized model used to derive DunedinPACNI included 99 regional brain measures. Because of difficulties in interpreting multivariable model coefficients, we used the Haufe transformation to estimate feature importance scores from the covariance between each brain measure and Pace of Aging^39^. Given that many of the MRI-derived measures are highly correlated, our elastic net model reduced overfitting by setting the weights for many of them to zero (visualized in Supplementary Fig. 1). Faster Pace of Aging covaried with thinner cortex, smaller cortical SA, smaller cortical GMV, lower cortical GWR, smaller subcortical GMV and larger ventricular volumes (Fig. 2c). We also observed positive covariance between calcarine CT and GWR, although not calcarine GMV. This is probably due to known aging-related effects on gray and white matter signal intensity that have been demonstrated previously^40–43^. These structural features overlap with the MRI signatures of both normal brain aging and neurodegenerative diseases^44–48^, suggesting that faster DunedinPACNI reflects, at least in part, canonical patterns of brain aging.

### DunedinPACNI has excellent test–retest reliability

If DunedinPACNI is to be used as a measure of aging, it must exhibit sufficient measurement reliability when exported to new datasets. We used test–retest MRI data (*n* = 45) from the HCP^49^ to estimate the reliability of DunedinPACNI. The test–retest reliability was excellent (intraclass correlation coefficient = 0.94, 95% CI = 0.89 to 0.97; Supplementary Fig. 2).

### DunedinPACNI is associated with worse cognitive functioning

**Fig. 3:**
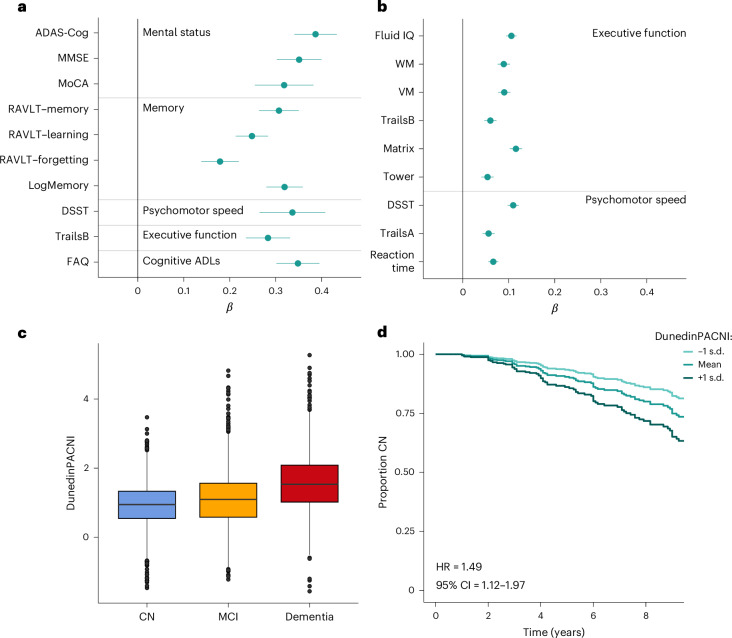
DunedinPACNI predicts cognition, cognitive impairment and conversion to dementia. **a**,**b**, Cross-sectional associations between DunedinPACNI and cognitive test scores in ADNI (**a**) and UKB (**b**). **a**,**b**, Effects are presented as standardized *β* coefficients with the error bars as the 95% CIs. We visualized the absolute effect sizes to aid visual comparison and clarity (see Supplementary Tables 2 and 3 for the raw effect sizes). The exact sample sizes for each test in **a** and **b** are reported in Supplementary Tables 2 and 3. **c**, Group differences in DunedinPACNI scores in 1,737 ADNI participants according to cognitive status at scanning. The center lines represent the median. The lower and upper hinges represent the 25th and 75th percentiles. The whiskers extend 1.5 times the interquartile range (IQR) from the hinges. Data beyond the whiskers are plotted as individual outliers. **d**, Survival curve of the relative proportion of CN ADNI participants at baseline who remained CN during the follow-up window, grouped according to slow, average and fast baseline DunedinPACNI scores. Note that although the maximum follow-up length is 16 years, we chose to visualize only 9 years of follow-up because of high amounts of censoring after 9 years. A plot with the full 16 years of follow-up and points marking censoring is presented in Extended Data Fig. 4. ADAS-Cog, Alzheimer’s Disease Assessment Scale-Cognitive Subscale 13; DSST, Digit Symbol Substitution Task; FAQ, Functional Activities Questionnaire; LogMemory, Logical Memory test; Matrix, Matrix Pattern Completion; MMSE, Mini-Mental State Examination; RAVLT, Rey Auditory Visual Learning Test; Tower, Tower Rearranging; TrailsA, Trail Making Test Part A; TrailsB, Trail Making Test Part B; VM, visual memory; WM, working memory. Source data

**Extended Data Fig. 3:**
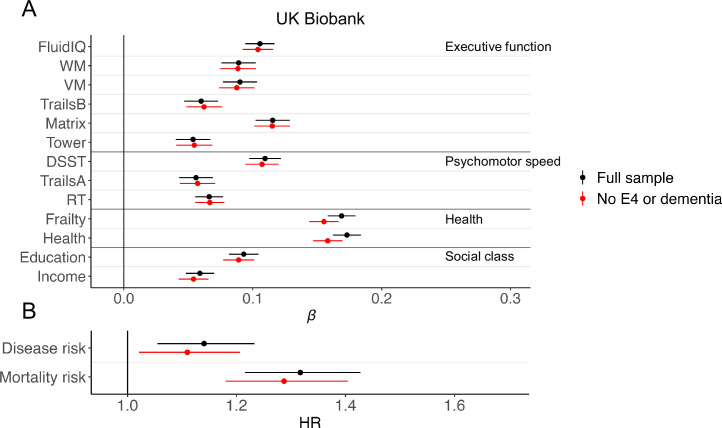
Associations with DunedinPACNI while excluding participants who go on to have cognitive decline or have high genetic risk for Alzheimer’s Disease in the UK Biobank. **a**. Forest plot of associations between cognitive, frailty, health, and social class measures and DunedinPACNI with and without inclusion of participants who later develop dementia or who carry two *APOE* E4 risk alleles. Effects are presented as standardized beta coefficients with error bars as 95% confidence intervals. Note that for visualization, the signs of some outcomes were flipped, such that higher scores for all outcomes reflected worse performance or health. **b**. Forest plot of DunedinPACNI hazard ratios for chronic disease and mortality with and without inclusion of participants who develop dementia or carry two *APOE* E4 risk alleles. Effects are presented as hazard ratios with error bars as 95% confidence intervals. Exact sample sizes for all tests are presented in Supplementary Table 4. Abbreviations: DSST = Digit Symbol Substitution Task, HR = hazard ratio, IQ = intelligence quotient, Matrix = Matrix Pattern Completion, RT = Reaction Time, Tower = Tower Rearranging, TrailsA = Trail Making Test Part A, TrailsB = Trail Making Test Part B, VM = Visual Memory, WM = Working Memory. Source data

Having established both internal validity and test–retest reliability, we sought to examine whether DunedinPACNI generalizes to new datasets to detect aging-related outcomes. Specifically, we first tested for associations with cognitive impairment in ADNI and cognitive functioning in UKB. We generated DunedinPACNI scores from T1-weighted MRI scans collected in 1,737 ADNI participants (mean age = 74.3 years, s.d. = 7.2, range = 52–97 years) and 42,583 UKB participants (mean age = 64.4 years, s.d. = 12.7, range = 44–82 years). In ADNI, participants with faster DunedinPACNI showed greater impairment on mental status examinations used to screen for dementia, as well as tests of memory, psychomotor speed and executive functioning. They also reported more impairment in cognitively demanding activities of daily living (ADLs), such as maintaining finances or preparing a meal (Fig. 3a). Absolute standardized effect sizes across all cognitive measures in ADNI ranged from *β* = 0.18 to 0.39 (all *P* < 0.001; full results in Supplementary Table 2). Similarly, UKB participants with faster DunedinPACNI performed more poorly on tests of executive functioning and psychomotor speed (Fig. 3b). Absolute standardized effect sizes across all cognitive measures in UKB ranged from *β* = 0.05 to 0.12 (all *P* < 0.001; full results in Supplementary Table 3). Associations in UKB were not driven by individuals with early cognitive decline or *APOE* ε4 homozygotes, who have heightened genetic risk for Alzheimer’s disease and show earlier onset of cognitive decline^50^ (Extended Data Fig. 3 and Supplementary Table 4). These, and all subsequent analyses presented in this article, control for sex and chronological age.

### DunedinPACNI predicts future cognitive decline and dementia

We next tested if DunedinPACNI differentiates between normal and clinically impaired cognitive functioning in ADNI (Fig. 3c). Participants with mild cognitive impairment (MCI) had faster DunedinPACNI compared to cognitively normal (CN) participants (*β* = 0.27, *P* < 0.001, 95% CI = 0.18 to 0.35). Participants with dementia had faster DunedinPACNI than both participants with MCI (*β* = 0.54, *P* < 0.001, 95% CI = 0.43 to 0.65) and CN participants (*β* = 0.81, *P* < 0.001, 95% CI = 0.69 to 0.92).

We further tested whether DunedinPACNI predicts future cognitive decline in people without cognitive impairment. Specifically, we analyzed a subsample of 624 ADNI participants who were CN at the time of their first scan (mean age = 72.4 years, s.d. = 6.3 years, range = 52.7–89.9 years), 112 of whom converted to either MCI or dementia during up to 16 years of follow-up (mean follow-up = 4.90 years). CN participants with faster DunedinPACNI at baseline were more likely to develop MCI or dementia and to do so earlier during the follow-up window (hazard ratio (HR) = 1.49, *P* = 0.005, 95% CI = 1.12 to 1.97; Fig. 3d), meaning that those in the top 10% had a 61% increased risk of developing MCI or dementia compared to participants with an average DunedinPACNI. We conducted a similar analysis in the 701 participants who were diagnosed with MCI at the time of their first scan (mean age = 72.8 years, s.d. = 7.3 years, range = 55.0–88.8 years), 271 of whom converted to dementia during up to 16 years of follow-up (mean follow-up = 4.10 years). Participants with MCI with faster DunedinPACNI at baseline were more likely to convert to dementia (HR = 1.44, *P* < 0.001, 95% CI = 1.26 to 1.65). These effect sizes were similar when controlling for the number of *APOE* ε4 alleles, a well-established genetic risk allele for sporadic, late-onset AD (baseline CN: HR = 1.49, *P* = 0.005, 95% CI = 1.13 to 1.96; baseline MCI: HR = 1.42, *P* < 0.001, 95% CI = 1.23 to 1.62). Because only a very small number of UKB participants with MRI data received diagnoses of dementia during the follow-up observation (*n* = 73), we were underpowered to report parallel results in this dataset.

### DunedinPACNI predicts accelerated brain atrophy

**Fig. 4:**
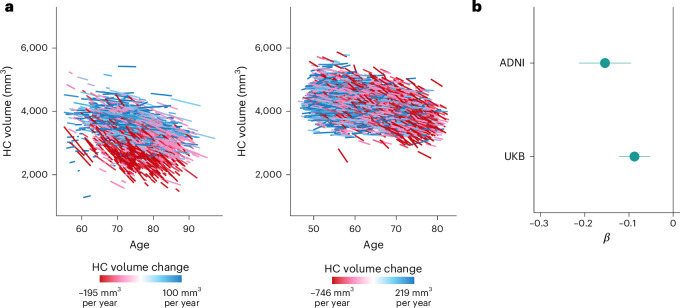
DunedinPACNI predicts accelerated hippocampal atrophy. **a**, Individualized trajectories of hippocampal atrophy in ADNI (left) and UKB (right). Warmer colors represent accelerated atrophy. **b**, Forest plot of associations between baseline DunedinPACNI scores and accelerated hippocampal atrophy in 1,302 ADNI participants and 4,601 UKB participants. Effects are presented as standardized *β* coefficients with the error bars as the 95% CIs. HC, hippocampus. Source data

As an estimate of how fast a person is aging, DunedinPACNI should reflect longitudinal trajectories of brain decline^33^. We tested whether faster baseline DunedinPACNI predicted accelerated hippocampal atrophy, which is an established risk factor for cognitive decline and dementia onset in older adults^51^. Specifically, we computed longitudinal trajectories of hippocampal atrophy among 1,302 ADNI participants who had MRI data at multiple time points (average number of scans = 4.4, range = 2–13 scans) as well as 4,601 UKB participants who had MRI data at two time points (Fig. 4a). Participants with faster baseline DunedinPACNI exhibited accelerated hippocampal atrophy in both ADNI (*β* = −0.15, *P* < 0.001, 95% CI = −0.21 to −0.10; Fig. 4b) and UKB (*β* = −0.09, *P* < 0.001, 95% CI = −0.12 to −0.05; Fig. 4b). This result was consistent while controlling for number of *APOE* ε4 alleles (Supplementary Table 5).

### DunedinPACNI predicts frailty, disease and mortality

As a measure of aging derived from longitudinal assessments of multiple biomarkers, DunedinPACNI should capture instances of declining health across all organ systems, not just the brain. To test this hypothesis, we used UKB to map DunedinPACNI scores onto measures of frailty, subjective overall health, incident aging-related chronic diseases and all-cause mortality.

**Fig. 5:**
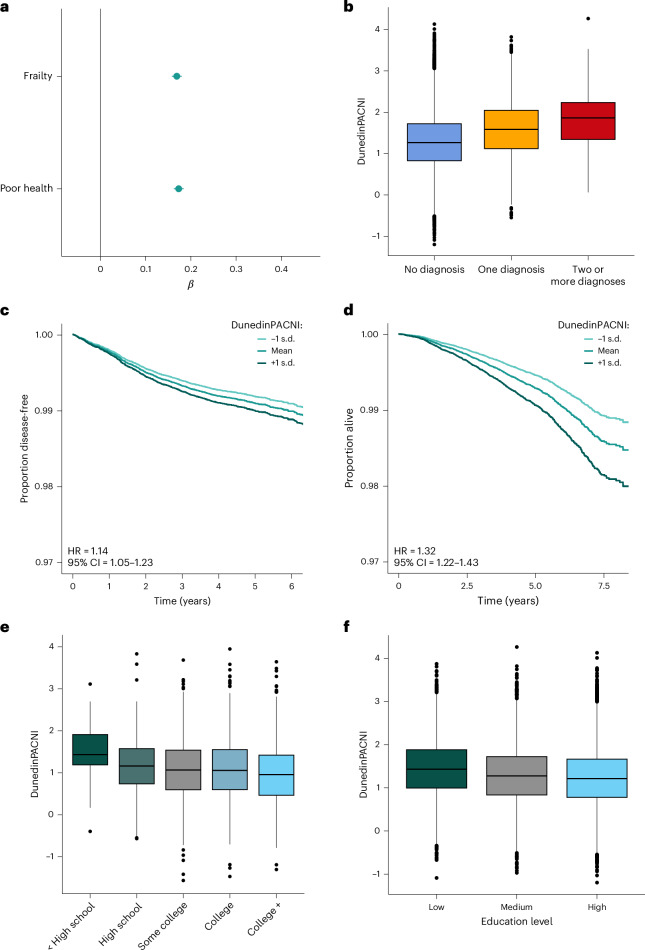
DunedinPACNI predicts frailty, poor health, multimorbidity, future chronic diseases and mortality, and reflects social gradients of health inequities. **a**, Forest plot of absolute associations between DunedinPACNI and frailty (*n* = 42,583) and self-rated health (*n* = 42,235) in UKB. Effects are presented as standardized *β* coefficients with the error bars as the 95% CIs. **b**, Group differences in DunedinPACNI scores according to the lifetime number of aging-related chronic disease diagnoses, including myocardial infarction, chronic obstructive pulmonary disease, dementia and stroke in 42,583 UKB participants. **c**, Survival curve of the relative proportion of disease-free UKB participants at the time of MRI who remained disease-free during the follow-up window, grouped according to slow, average and fast baseline DunedinPACNI scores. We excluded participants who had chronic disease before scanning from this analysis. **d**, Survival curve of the relative proportion of UKB participants who remained alive during the follow-up window grouped according to baseline DunedinPACNI scores. **e**, Group differences in DunedinPACNI according to education level in 1,734 ADNI participants. **f**, Group differences in DunedinPACNI according to education level in 38,297 UKB participants. **b**,**e**,**f**, The center lines represent the median. The lower and upper hinges represent the 25th and 75th percentiles. The whiskers extend 1.5 times the IQR from the hinges. Data beyond the whiskers are plotted as individual outliers. Source data

We used the Fried Frailty Index to quantify the degree of vulnerability to common stressors associated with aging-related decline in energy reserves and functioning. When treating index scores as a continuous measure ranging from 0 to 5, with higher scores indicating greater frailty^52,53^, we found that participants with faster DunedinPACNI were frailer (*n* = 42,583; *β* = 0.17, *P* < 0.001, 95% CI = 0.16 to 0.18). Participants with faster DunedinPACNI also self-reported poorer overall health (*n* = 42,235; *β* = −0.17, *P* < 0.001, 95% CI = −0.18 to −0.16; Fig. 5a), which predicts mortality even independently of objective health measures^54^. These associations were not driven by individuals with early cognitive decline or high genetic risk for AD (Extended Data Fig. 3 and Supplementary Table 4).

Similar patterns emerged when considering clinical diagnoses of chronic aging-related diseases, including myocardial infarction, chronic obstructive pulmonary disease, dementia and stroke. Participants with a lifetime prevalence of one of these chronic diseases had faster DunedinPACNI compared to those with no diagnoses (*β* = 0.19, *P* < 0.001, 95% CI = 0.16 to 0.23). Participants with a lifetime prevalence of two or more chronic diseases had faster DunedinPACNI than those with a single chronic disease (*β* = 0.25, *P* < 0.001, 95% CI = 0.12 to 0.38) and those with no chronic disease (*β* = 0.44, *P* < 0.001, 95% CI = 0.31 to 0.57; Fig. 5b).

Extending beyond contemporaneous associations, we assessed whether faster DunedinPACNI at baseline predicted future myocardial infarction, chronic obstructive pulmonary disease, dementia or stroke in UKB participants who were diagnosis-free at the time of scanning (*n* = 40,753). A total of 827 participants reported a new diagnosis of at least one of these aging-related chronic diseases over a maximum follow-up period of 9.7 years after scanning (that is, baseline). Consistent with the contemporaneous associations, healthy participants with faster DunedinPACNI at baseline were more likely to be later diagnosed with chronic aging-related diseases (HR = 1.14, *P* < 0.001, 95% CI = 1.05 to 1.23; Fig. 5c), that is, those in the top 10% had an 18% or greater increased risk of developing a chronic disease compared to participants with average DunedinPACNI. These associations were not driven by individuals with early cognitive decline or high genetic risk for AD (Extended Data Fig. 3 and Supplementary Table 4).

Given the increased mortality rates among people with chronic aging-related diseases, we asked if baseline DunedinPACNI scores predicted all-cause mortality. Of the 42,583 UKB participants included in our dataset, 757 died over the follow-up period after their baseline MRI scan. Participants with faster baseline DunedinPACNI scores died earlier (HR = 1.32, *P* < 0.001, 95% CI = 1.22 to 1.43; Fig. 5d), that is, those in the top 10% were at least 41% more likely to die compared to participants with average DunedinPACNI. These associations were not driven by individuals with early cognitive decline or high genetic risk for AD (Extended Data Fig. 3 and Supplementary Table 4). Taken together, these findings suggest that DunedinPACNI is useful for gauging general physical health and assessing the risk for future chronic disease and death.

### DunedinPACNI reflects social gradients of health inequities

People who are less advantaged in their socioeconomic position experience a wide range of chronic diseases and earlier mortality^55–57^; DunedinPACNI should reflect such gradients of health inequities. We used information about educational attainment and income to test this prediction. Faster DunedinPACNI was observed for participants who either had fewer years of formal education (ADNI: *β* = −0.10, *P* < 0.001, 95% CI = −0.15 to −0.05]; UK Biobank: *β* = −0.09, *P* < 0.001, 95% CI = −0.10 to −0.08) or lower income (UKB: *β* = −0.06, *P* < 0.001, 95% CI = −0.07 to −0.05), reflecting the expected socioeconomic health gradient (Fig. 5e,f). These associations were not driven by individuals with early cognitive decline or high genetic risk for AD (Extended Data Fig. 3).

### DunedinPACNI generalizes to a Latin American sample

**Fig. 6:**
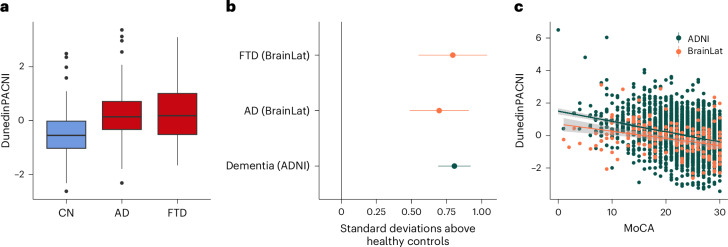
DunedinPACNI is similarly associated with dementia and cognitive impairment in a sample of BrainLat participants. **a**, Group differences in DunedinPACNI scores according to cognitive diagnosis in 369 BrainLat participants. The center lines represent the median. The lower and upper hinges represent the 25th and 75th percentiles. The whiskers extend 1.5 times the IQR from the hinges. Data beyond the whiskers are plotted as individual outliers. **b**, Forest plot of standardized mean differences in DunedinPACNI between participants with dementia and CN controls in BrainLat (orange; *n* = 369) and ADNI (dark green; *n* = 1,201) while controlling for age and sex. DunedinPACNI was similarly accelerated in dementia in a sample of Latin Americans (BrainLat) and North Americans (ADNI). Effects are presented as standardized *β* coefficients with the error bars as the 95% CIs. **c**, Scatter plot of associations between MoCA scores and DunedinPACNI in BrainLat (orange; *n* = 191) and ADNI (dark green, *n* = 1,206) participants. The regression error bands represent the 95% CIs. DunedinPACNI scores were residualized for age and sex. The linear associations between MoCA scores and DunedinPACNI scores were similar in a sample of Latin Americans (BrainLat) and North Americans (ADNI). Source data

Brain-based predictive algorithms often fail when applied to groups that demographically differ from the training sample^58^. Most cognitive and brain aging studies are collected in high-income countries in North America and Europe; this may limit the generalizability of brain-based models to people in low-income and middle-income countries^59–61^. Latin Americans are underrepresented in biomedical research^60,62^ and may experience distinct social and environmental influences on brain aging compared to people in high-income countries in North America and Europe^60^. To test the generalizability of DunedinPACNI in Latin Americans, we calculated DunedinPACNI scores in a sample of 369 adults from Argentina, Chile, Colombia, Mexico and Peru from the BrainLat dataset^63^. A total of 162 BrainLat participants were diagnosed with AD, 84 were diagnosed with behavioral variant frontotemporal dementia (FTD) and 123 were healthy controls. When controlling for age and sex, BrainLat participants with AD and FTD had faster DunedinPACNI scores compared to healthy controls (AD: *β* = 0.70, *P* < 0.001, 95% CI = 0.48 to 0.91; FTD: *β* = 0.79, *P* < 0.001, 95% CI = 0.55 to 1.04; Fig. 6a). Notably, these effect sizes are comparable to the difference between participants with dementia and CN participants in ADNI (Fig. 6b). In addition, 191 BrainLat participants also completed the Montreal Cognitive Assessment (MoCA). In this subset, BrainLat participants with faster DunedinPACNI scores had poorer scores on the MoCA (*β* = −0.35, *P* < 0.001, 95% CI = −0.49 to −0.20). Notably, this effect was similar to the association between DunedinPACNI and MoCA scores in ADNI participants (*β* = −0.32, *P* < 0.001, 95% CI = −0.38 to −0.25; Fig. 6c).

### DunedinPACNI is distinct from common measures of brain aging

**Fig. 7:**
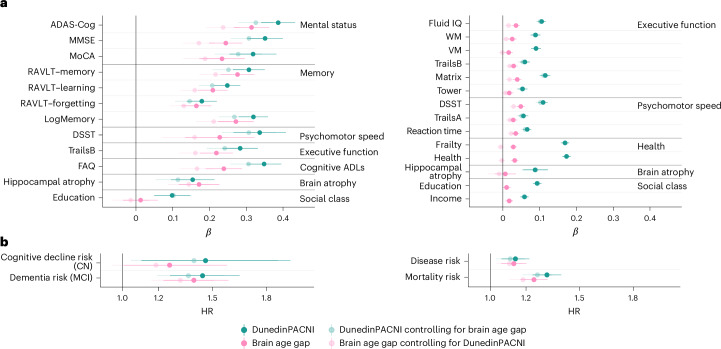
Comparison of DunedinPACNI and brain age gap associations with aging-related phenotypes. **a**, Forest plots of DunedinPACNI and brain age gap absolute effect sizes in ADNI (left) and UKB (right). Effects are presented as standardized *β* coefficients with the error bars as the 95% CIs. Note that for visualization, the signs of some outcomes were flipped, such that higher scores for all outcomes reflected worse performance or health. Raw effect sizes are presented in Supplementary Tables 2, 3, 7 and 9. **b**, Forest plots of DunedinPACNI and brain age gap HRs in ADNI and UKB. Effects are presented as HRs with the error bars as the 95% CIs. **a**,**b**, Exact sample sizes for each test are reported in Supplementary Tables 2, 3 and 7–9). Lighter shades represent the effect size for each measure while controlling for the other measure (that is, the effect of DunedinPACNI when controlling for brain age gap and vice versa). ADAS-Cog, Alzheimer’s Disease Assessment Scale-Cognitive Subscale 13; DSST, Digit Symbol Substitution Task; FAQ, Functional Activities Questionnaire; LogMemory, Logical Memory test; Matrix, Matrix Pattern Completion; MMSE, Mini-Mental State Examination; RAVLT, Rey Auditory Visual Learning Test; Tower, Tower Rearranging; TrailsA, Trail Making Test Part A; TrailsB, Trail Making Test Part B; VM, visual memory; WM, working memory. Source data

**Extended Data Fig. 5:**
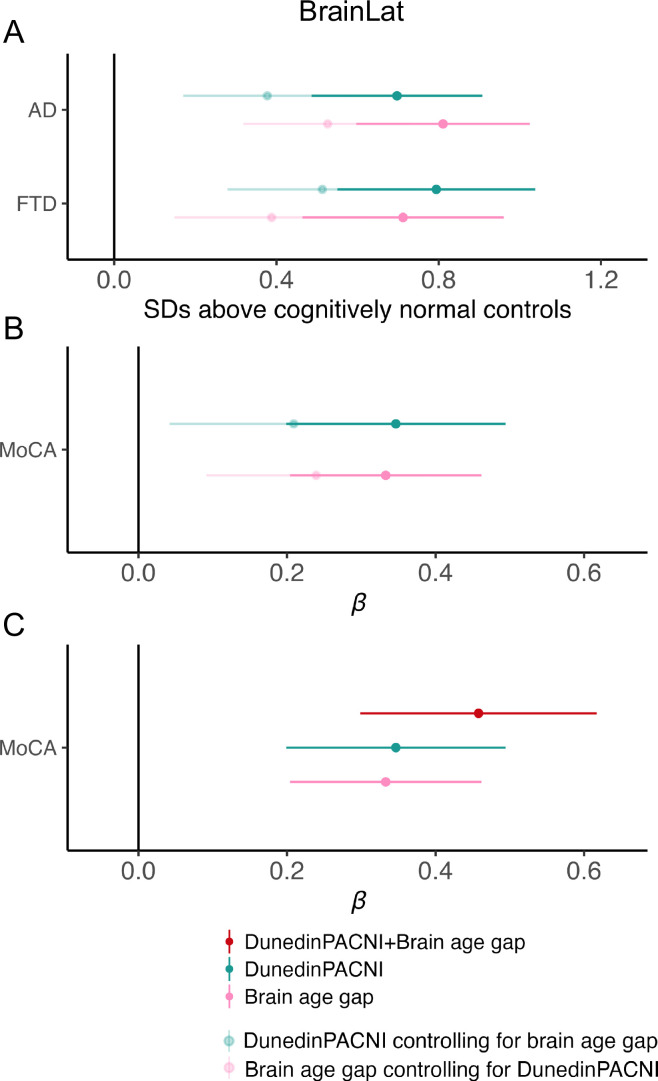
Comparison of DunedinPACNI and brain age gap associations with clinical outcomes in BrainLat. **a**. Forest plots of DunedinPACNI and brain age gap standardized mean differences between dementia groups and healthy controls in BrainLat. Error bars represent 95% confidence intervals. Lighter shades represent the effect size for each measure while controlling for the other measure (that is, effect of DunedinPACNI when controlling for brain age gap, and vice versa). **b**. Forest plots of DunedinPACNI and brain age gap absolute standardized association effect sizes with the MoCA. Error bars represent 95% confidence intervals. Lighter shades represent the effect size for each measure while controlling for the other measure (that is, effect of DunedinPACNI when controlling for brain age gap, and vice versa). **c**. Forest plot of absolute standardized associations between DunedinPACNI alone, brain age gap alone, and their combined associations with MoCA scores in BrainLat. Error bars represent 95% confidence intervals. Exact sample sizes for all tests are presented in Supplementary Tables 10, 11). Abbreviations: AD = Alzheimer’s dementia, FTD = frontotemporal dementia, MoCA = Montreal Cognitive Assessment, SDs = standard deviations. Source data

**Extended Data Fig. 6:**
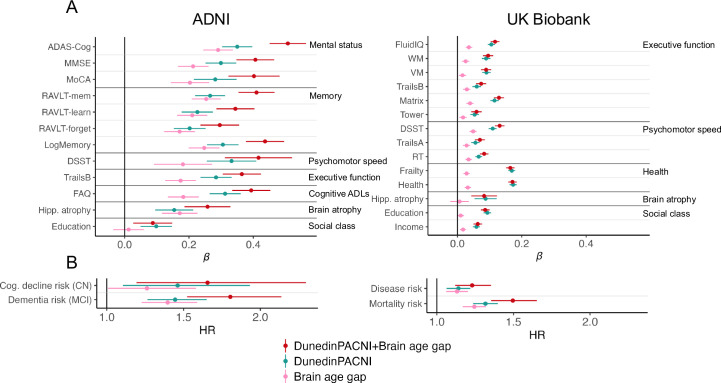
Combined effects of DunedinPACNI and brain age gap were generally more sensitive to clinical outcomes. **a**. Forest plots for effect sizes for DunedinPACNI alone, brain age gap alone, and their combined associations with clinical outcomes. Combined effects were considered to be the sum of beta coefficients when both DunedinPACNI and brain age gap were included in a model while controlling for age and sex. Error bars represent 95% confidence intervals. Note that for visualization, the signs of some outcomes were flipped, such that higher scores for all outcomes reflected worse performance or health. **b**. Forest plots of hazard ratios for risk of cognitive decline, dementia, chronic disease, and death. Combined effects were considered to be the products of respective hazard ratios for DunedinPACNI and brain age gap included in a model while controling for age and sex. Error bars represent 95% confidence intervals. Exact sample sizes for each test in **a**. and **b**. are reported in Supplementary Tables 2, 3, 7–9). Note that for cognitive tests in ADNI, only baseline observations were used in this analysis to facilitate combination of effects. Thus, sample sizes for these tests are reflected by the number of individuals, not observations, in the sample. This information is provided in Supplementary Table 2. Abbreviations: ADAS-Cog = Alzheimer’s Disease Assessment Scale – Cognitive Subscale 13, ADLs = activities of daily living, ADNI = Alzheimer’s Disease Neuroimaging Initiative, CI = confidence interval, CN = cognitively normal, Cog. = cognitive, DSST = Digit Symbol Substitution Task, FAQ = Functional Activities Questionnaire, Hipp. = hippocampal, HR = hazard ratio, IQ = intelligence quotient, LogMemory = Logical Memory, Matrix = Matrix Pattern Completion, MCI = mild cognitive impairment, MMSE = Mini-Mental State Exam, MoCA = Montreal Cognitive Assessment, RT = Reaction Time, RAVLT = Rey Auditory Visual Learning Test, SD = standard deviation, Tower = Tower Rearranging, TrailsA = Trail Making Test Part A, TrailsB = Trail Making Test Part B, VM = Visual Memory, WM = Working Memory. Source data

Lastly, we compared DunedinPACNI with existing approaches for measuring aging using brain MRI data. Specifically, we compared the effect sizes for DunedinPACNI from all of the aforementioned analyses in ADNI, UKB and BrainLat with brain age gap generated using brainageR^34^. We selected this algorithm because of its high accuracy and test–retest reliability compared to other brain age gap algorithms^64^. DunedinPACNI and brain age gap were only modestly correlated (ADNI: *r* = 0.17, *P* < 0.001; UKB: *r* = 0.31, *P* < 0.001; BrainLat: *r* = 0.32, *P* < 0.001; Supplementary Fig. 3). Compared to brain age gap, the effect sizes for DunedinPACNI were similar or larger across measures of cognitive function, cognitive decline, brain atrophy, frailty, disease risk, mortality and socioeconomic health gradients (Fig. 7 and Extended Data Figs. 5 and 6; full results are shown in Supplementary Tables 2, 3 and 6–11). Commensurate with the low correlation between these measures, when we included both DunedinPACNI and brain age gap in a single model, each measure explained unique variance in clinical outcomes, with only minor reductions in effect sizes. Moreover, using both DunedinPACNI and brain age gap together in a single model generally increased the prediction of these outcomes (Extended Data Figs. 5 and 6). For example, the combined HR of DunedinPACNI and brain age gap predicting mortality risk was 1.50 (95% CI = 1.36 to 1.65), compared to the independent HRs of 1.32 for DunedinPACNI and 1.24 for brain age gap.

**Extended Data Fig. 7:**
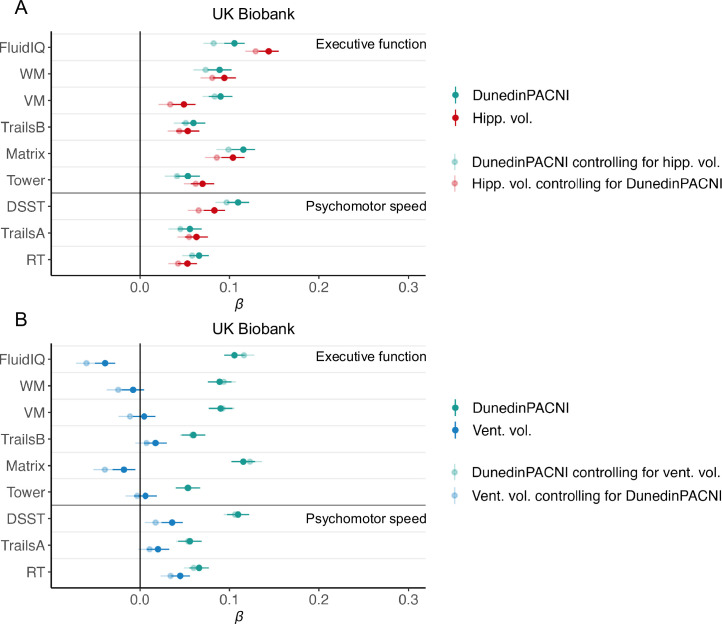
Cross-sectional cognitive associations with DunedinPACNI, hippocampal volume, and ventricular volume in the UK Biobank. **a**. Forest plot of associations between cognitive measures and DunedinPACNI and hippocampal volume. Effects are presented as standardized beta coefficients with error bars as 95% confidence intervals. Note that we flipped the direction of hippocampal associations to match the direction of DunedinPACNI. **b**. Forest plot of associations between cognitive measures and DunedinPACNI and ventricular volume. Effects are presented as standardized beta coefficients with error bars as 95% confidence intervals. Lighter shades represent the effect size for each measure while controlling for the other measure (that is, effect of DunedinPACNI when controlling for hippocampal or ventricular volume, and vice versa). Note that for visualization, the signs of some outcomes were flipped, such that higher scores for all outcomes reflected worse performance or health. Exact effect sizes and sample sizes for each test are presented in Supplementary Table 12, 13). Abbreviations: DSST = Digit Symbol Substitution Task, Hipp. = hippocampus, IQ = intelligence quotient, Matrix = Matrix Pattern Completion, RT = Reaction Time, Tower = Tower Rearranging, TrailsA = Trail Making Test Part A, TrailsB = Trail Making Test Part B, Vent. = ventricle, vol. = volume, VM = Visual Memory, WM = Working Memory. Source data

**Extended Data Fig. 8:**
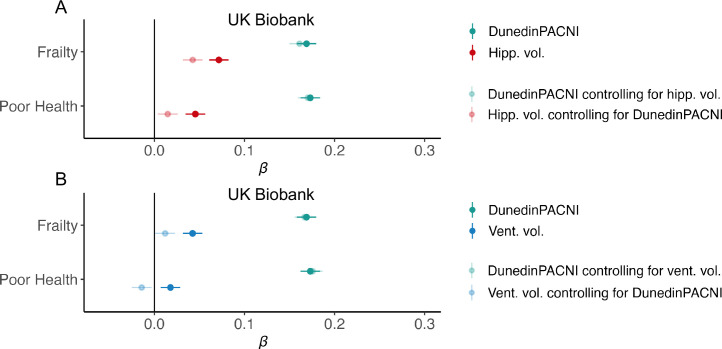
Cross-sectional frailty and health associations with DunedinPACNI, hippocampal volume, and ventricular volume in the UK Biobank. **a**. Forest plot of associations between frailty and health measures and DunedinPACNI and hippocampal volume. Effects are presented as standardized beta coefficients with error bars as 95% confidence intervals. Note that we flipped the direction of hippocampal associations to match the direction of DunedinPACNI. **b**. Forest plot of associations between frailty and health measures and DunedinPACNI and ventricle volume. Effects are presented as standardized beta coefficients with error bars as 95% confidence intervals. Lighter shades represent the effect size for each measure while controlling for the other measure (that is, effect of DunedinPACNI when controlling for hippocampal or ventricular volume, and vice versa). Exact sample sizes for each test are presented in Supplementary Table 12, 13). Abbreviations: Hipp. = hippocampus, Vent. = ventricle, vol. = volume. Source data

**Extended Data Fig. 9:**
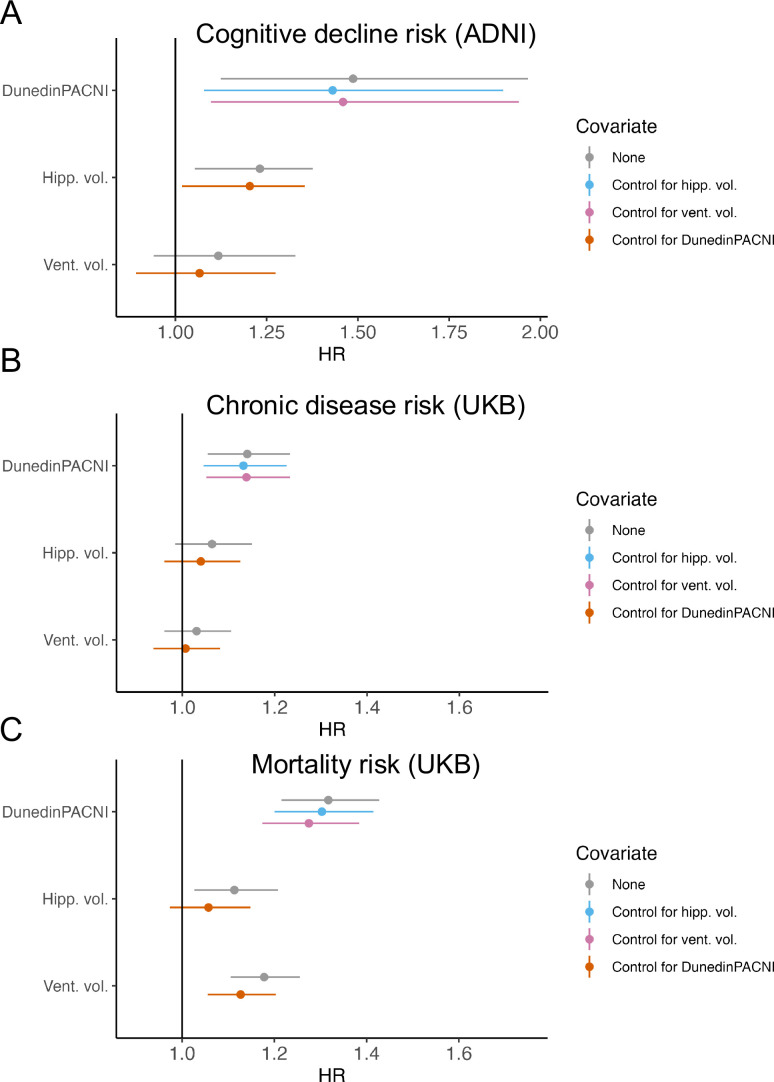
Cognitive decline, disease, and mortality prediction by DunedinPACNI, hippocampal volume, and ventricular volume. **a**. Cognitive decline prediction among cognitively normal ADNI participants, **b**. Chronic disease prediction in UK Biobank. **C**. Mortality prediction in UK Biobank. Gray points indicate hazard ratios for each brain measure while covarying for sex and age at scan. The blue point indicates the DunedinPACNI hazard ratio while also covarying for hippocampal volume. The pink point indicates the DunedinPACNI hazard ratio while covarying for ventricular volume. The orange points indicate the hippocampal and ventricular volume hazard ratios while covarying for DunedinPACNI. Note that we flipped the direction of hippocampal associations to match the direction of DunedinPACNI. Error bars on all plots represent 95% confidence intervals. Exact sample sizes for each test are presented in Supplementary Table 14). Abbreviations: ADNI = Alzheimer’s Disease Neuroimaging Initiative, Hipp. = hippocampus, HR = hazard ratio, UKB = UK Biobank, Vent. = ventricle, vol. = volume. Source data

In addition, we investigated how DunedinPACNI differs from commonly reported MRI-based measures of brain aging, namely hippocampal volume and ventricular volume. To test this, we compared the effect sizes for DunedinPACNI with the effect sizes for bilateral hippocampal volume and bilateral ventricular volume in UKB and in CN participants in ADNI. In both datasets, we observed that a faster DunedinPACNI was generally more strongly and more consistently associated with poor cognition, poor health and frailty, as well as risk of dementia, disease and mortality. Furthermore, DunedinPACNI explained incremental variance in these outcomes over and above hippocampal volume and ventricular volume alone (Extended Data Figs. 7–9 and Supplementary Tables 12–14).

## Discussion

DunedinPACNI is an accurate and reliable measure of how fast a person is aging derived from a single brain MRI scan. Using 50,106 scans from people ranging from 22 to 98 years old across the HCP, ADNI, UKB and BrainLat datasets, we demonstrate that people with faster DunedinPACNI had not only worse cognitive and brain health (that is, poorer cognition, faster hippocampal atrophy and greater dementia risk) but also worse general health (that is, greater frailty, poorer self-reported health, greater risk of chronic disease and mortality). This indicates that patterns of aging detected during midlife are clinically useful among people in advanced age, including people with neurodegenerative disease. Furthermore, DunedinPACNI showed evidence of generalization to a sample of Latin American adults with and without dementia. Across all analyses, the effect sizes for DunedinPACNI were similar or larger than the effect sizes for brain age gap, an existing age deviation measure estimated using the same structural MRI data. Moreover, DunedinPACNI and brain age gap were only weakly correlated, and DunedinPACNI accounted for incremental variance in aging-related health outcomes. While weak correlations between neuroimaging-based measures of aging may appear surprising, they mirror findings that different epigenetic clocks are also weakly correlated, and that multiple clocks are useful for predicting disease and death^65,66^. It is possible that biomarkers of aging trained in midlife samples more closely detect the earlier stages of aging compared to biomarkers of aging trained in samples of advanced age. This may partially explain the modest correlations observed between aging biomarkers. Aging remains a construct in search of measurement tools^4,5^, and DunedinPACNI represents a next-generation measure of aging that is distinct from existing approaches.

DunedinPACNI is not without limitations. First, the Dunedin Study, ADNI and UKB consist of data collected primarily from participants of European ancestry. Furthermore, ADNI and UKB oversample participants from higher socioeconomic backgrounds^67^. There is growing awareness that lack of representativeness in neuroimaging research may hinder clinical translation^68,69^, including clinical translation of brain-based predictive models^58^. To address this, we replicated associations with DunedinPACNI in a sample of Latin Americans, as well as low-income and non-White UKB participants. (Supplementary Tables 15 and 16). Additionally, our findings in ADNI, UKB and BrainLat demonstrate that DunedinPACNI generalizes well to older adults. This suggests that early differences in aging can be detected in brain MRI measures collected at age 45 years that are clinically useful in people who are decades older. At the same time, it is also possible that an analogous aging biomarker trained in older adults may generalize more readily to other samples of older adults. A priority for future work is to further evaluate the generalizability of DunedinPACNI to people of diverse demographics and backgrounds. Second, DunedinPACNI only uses structural brain measures derived from a T1-weighted MRI scan. We chose this strategy because these scans are collected in nearly every MRI study, thereby maximizing the potential adoption of DunedinPACNI. It is possible that the performance accuracy reported in this study could be improved by including additional structural or functional MRI measures (for example, white matter microstructural integrity from diffusion-weighted images, blood-oxygen-level-dependent signal from T2*-weighted images). Relatedly, while elastic net is widely used to measure biological age^11,12,15,16,20^, future research should evaluate whether predictive performance accuracy could be increased by more complex, nonlinear models. Third, by design, DunedinPACNI is a measure of the longitudinal rate of the aging of the body derived from a single MRI scan and is not designed to replace longitudinal measurement of brain aging through repeated MRI assessments. Fourth, DunedinPACNI is estimated through observed correlations between measures of brain structure and longitudinal aging, which could reflect multiple causal pathways. For example, faster aging of non-brain organs might cause poorer brain health or vice versa. Alternatively, both may be driven by a third factor. Fifth, although we found robust associations with aging phenotypes across both ADNI and UKB, we generally observed larger effect sizes in ADNI. This could suggest that DunedinPACNI is sensitive to dysfunction in individuals with neurodegenerative diseases. Further evaluation is needed to establish the degree to which DunedinPACNI is sensitive to individual organ systems. Sixth, DunedinPACNI is currently a quantitative tool for comparison of individuals within datasets but not between datasets. Future research, potentially through data harmonization and normative modeling approaches, should establish normative reference values and ranges that can aid in the clinical interpretation of DunedinPACNI scores. Seventh, as is true for all MRI-based measurements, the derivation of DunedinPACNI may be compromised in low-quality scans, such as those with high levels of head motion. In the analyses presented in this study, we followed standard practice by excluding low-quality scans (Supplementary Figs. 4–7). Future research should adopt methods to improve data quality in high-motion participants to help maximize generalizability^70,71^.

Currently, DunedinPACNI and general aging biomarkers are research tools that require further validation before potential translation to the clinic^5^. Nonetheless, we believe that DunedinPACNI and other biomarkers of general aging have several promising uses that differ from, and are complementary to, biomarkers of specific diseases (for example, hippocampal atrophy and AD^72^ or high blood pressure and stroke^73^). For example, biomarkers of general aging could help identify a broad risk for many age-related diseases earlier in the lifespan, when individuals may benefit most from health and antiaging intervention^23^. Biomarkers of general aging could also help establish a person’s prognosis after the onset of a specific disease or establish how interventions or insults may change how fast a person is aging^25,74,75^. Lastly, biomarkers of general aging are necessary to mechanistically link exposures (for example, low socioeconomic status) to aging or to link the rate of aging to outcomes such as increased disease risk^4^. While detecting or diagnosing a specific disease requires a proximal, specific biomarker of that disease, biomarkers of general aging could be used to measure the distal risk for a broad array of diseases. This difference allows for clinical applications of biomarkers of aging such as DunedinPACNI alongside biomarkers of specific diseases.

Several unique features of the Dunedin Study contribute advantages to DunedinPACNI compared to other biomarkers of aging. First, DunedinPACNI was developed in a cohort of people all born in the same year and studied at the same ages throughout their lives, thereby avoiding biases that are introduced by differences in historical exposures across generations and across time. Second, DunedinPACNI was trained on 19 biomarkers that were each assessed over nearly two decades and thus are not influenced by short-term illnesses that can cause aberrant biomarker signals at a single assessment. Third, DunedinPACNI was derived from participants followed from birth to age 45 years—before the onset of chronic, aging-related diseases that cause divergence from typical trajectories of aging. Other biomarkers of aging are typically derived from multiage samples in which many of the older members already have chronic diseases that have altered their body and brain, so those measures may inadvertently repackage disease instead of aging. Fourth, because the Dunedin Study cohort is a population representative cohort with very low attrition and mortality rates, DunedinPACNI does not suffer from oversampling of healthy volunteers, attrition bias (that is, people with worse health being more likely to drop out) or survivor bias (that is, people with worse health dying earlier). Indeed, our results align with prior research showing that DunedinPACE, which like DunedinPACNI was trained on the longitudinal Pace of Aging, is associated with dementia, morbidity and mortality^20,22,23^. Our results, alongside the fast-growing literature on DunedinPACE, suggest that these unique design characteristics of the Dunedin Study make it a powerful training sample for longitudinal biomarkers of aging.

The scope of geroscience has rapidly expanded with the proliferation of omic clocks that can measure how fast people age^10^. DunedinPACNI is poised to further this growth by allowing individual differences in the rate of longitudinal aging to be estimated from a single noninvasive MRI scan that can be collected in just a few minutes. Indeed, the requisite MRI data to estimate DunedinPACNI have already been collected in many psychiatric, neurological and brain health cohorts, from tens of thousands of research participants across the lifespan and around the world. DunedinPACNI offers an opportunity to enrich such studies and deepen our understanding of the causes of individual differences in the rate of longitudinal aging, including genetics^76^, childhood adversities^77^, environmental exposures (for example, lead^78,79^) and lifestyle factors (for example, physical inactivity, social isolation^80^). DunedinPACNI may also be adopted as a surrogate endpoint to accelerate our ability to develop, prioritize and evaluate potential antiaging interventions that slow aging and prevent disease^81,82^. The algorithm for DunedinPACNI is publicly available to the research community to facilitate these and other future research directions (https://github.com/etw11/DunedinPACNI).

## Methods

This research complies with all relevant ethical regulations; all study protocols were approved by the relevant ethical review boards. The specific ethical review boards are detailed in the description of each dataset. The premise and analysis plan for this study were preregistered (https://rb.gy/b9x4u6). All analyses and code were checked for accuracy by an independent analyst. Analyses were conducted on data collected through the Dunedin Study, HCP, ADNI, UKB and BrainLat. The details of each study and dataset are described below.

### Data sources

#### Dunedin Study

Participants are members of the Dunedin Study, a longitudinal investigation of health and behavior in a population-representative birth cohort. The 1,037 participants (91% of eligible births, 48% female) were all people born between April 1972 and March 1973 in Dunedin, New Zealand, who were residents in the province and who participated in the first assessment at age 3 years^19^. The cohort represented the full range of socioeconomic status in the general population of New Zealand’s South Island and, as adults, matched the New Zealand National Health and Nutrition Survey on key adult health indicators (for example, BMI, smoking and general practitioner visits) and the New Zealand Census of citizens of the same age on educational attainment^19,83^. Study members are primarily of New Zealand European ethnicity; 8.6% reported Māori ethnicity at age 45 years.

General assessments were performed at birth as well as ages 3, 5, 7, 9, 11, 13, 15, 18, 21, 26, 32 and 38 years, and most recently (completed April 2019) at age 45 years, when 938 of the 997 living study members (94.1%) participated. At each assessment, study members were brought to the Dunedin Study Research Unit at the University of Otago for interviews and examinations. In addition, staff provided standardized ratings; informant questionnaires were sent to people who the study members nominated as people who knew them well, and administrative records were searched. The Dunedin Study was approved by the University of Otago Ethics Committee and study members gave written informed consent before participating.

##### MRI

As a component of the assessments at age 45 years, study members were scanned using a Siemens MAGNETOM Skyra (Siemens Healthineers) 3T scanner equipped with a 64-channel head/neck coil at the Pacific Radiology Group imaging center in Dunedin, New Zealand. High-resolution T1-weighted images were obtained using an MP-RAGE sequence with the following parameters: repetition time (TR) = 2,400 ms; echo time (TE) = 1.98 ms; 208 sagittal slices; flip angle, 9°; field of view (FOV), 224 mm; matrix = 256 × 256 px; slice thickness = 0.9 mm with no gap (voxel size 0.9 × 0.875 × 0.875 mm); and total scan time = 6 min and 52 s. Three-dimensional (3D) fluid-attenuated inversion recovery (FLAIR) images were obtained with the following parameters: TR = 8,000 ms; TE = 399 ms; 160 sagittal slices; FOV = 240 mm; matrix = 232 × 256 px; slice thickness = 1.2 mm (voxel size 0.9 × 0.9 × 1.2 mm); and total scan time = 5 min and 38 s. Additionally, a gradient echo field map was acquired with the following parameters: TR = 712 ms; TE = 4.92 and 7.38 ms; 72 axial slices; FOV = 200 mm; matrix = 100 × 100 px; slice thickness = 2.0 mm (voxel size 2 mm isotropic); and total scan time = 2 min and 25 s. Of the 938 study members seen at phase 45, 63 declined to participate in MRI scanning, that is, 875 study members completed the MRI scanning protocol. Scanned study members did not differ from other living participants in terms of childhood neurocognitive functioning or childhood socioeconomic status (see the attrition analysis in Extended Data Figs. 1 and 2). Of these 875 study members for whom data was available, four were excluded because of major incidental findings or previous injuries (for example, large tumors or extensive damage to the brain or skull), nine because of missing FLAIR or field map scans, one because of poor surface mapping yielding and one because of missing the Pace of Aging variable. This yielded a final training sample of 860 study members (see Supplementary Fig. 4 for the inclusion details).

Structural MRI data were processed using FreeSurfer v.6.0 (ref. ^36^). Specifically, T1-weighted images were processed and refined with 3D FLAIR images using the recon-all pipeline.

##### Pace of Aging

Participants’ pace of biological aging was measured as changes in 19 biomarkers of study members’ cardiovascular, metabolic, pulmonary, kidney, immune and dental systems across ages 26, 32, 38 and 45 years. This measure quantifies participants’ rate of aging in year-equivalent units of physiological decline per chronological year. The average participant experienced 1 year of physiological decline per year, that is, a mean (s.d.) Pace of Aging of 1 (0.3)^2^. See the ‘Statistical analysis’ section for more details.

##### Physical functioning

One-legged balance was measured using the unipedal stance test as the maximum time achieved across three trials of the test with eyes closed^84–86^. Gait speed (meters per second) was assessed with the 6-m-long GAITRite Electronic Walkway (CIR Systems) with 2-m acceleration and 2-m deceleration before and after the walkway, respectively. Gait speed was assessed under three walking conditions: usual gait speed (walk at a normal pace from a standing start, measured as a mean of two walks) and two challenge paradigms, dual-task gait speed (walk at a normal pace while reciting alternate letters of the alphabet out loud, starting with the letter ‘A’, measured as a mean of two walks) and maximum gait speed (walk as fast as safely possible, measured as a mean of three walks). Gait speed was correlated across the three walk conditions^87^. To increase reliability and take advantage of the variation in all three walk conditions (usual gait and the two challenge paradigms), we calculated the mean of the three highly correlated individual walk conditions to generate our primary measure of composite gait speed. The step in place test was measured as the number of times the right knee was lifted to mid-thigh height (measured as the height half-way between the knee cap and the iliac crest) in 2 min at a self-directed pace^88,89^. Chair rises were measured as the number of stands with no hands completed in 30 s from a seated position^88,90^. Handgrip strength was measured for each hand (elbow held at 90°, upper arm held tight against the trunk) as the maximum value achieved across three trials using a Jamar digital dynamometer^91,92^. Analyses using handgrip strength controlled for BMI. Visuomotor coordination was measured as the time to completion of the grooved pegboard test. Scores were reversed so that higher values corresponded to better performance. Physical limitations were measured with the RAND 36-Item Health Survey 1.0 physical functioning scale. Responses (‘limited a lot’, ‘limited a little’, ‘not limited at all’) assessed difficulty with completing several activities (for example, climbing several flights of stairs, walking more than 1 km, participating in strenuous sports). Scores were reversed to reflect physical limitations so that a high score indicates more limitations.

##### Subjective health and age appearance

We obtained reports about study members’ health and age appearance from three sources: self-reports; informant impressions; and staff impressions. For self-reports, we asked study members about their own impressions of how old they looked: ‘Do you think you look older, younger, or about your actual age?’ Response options were younger than their age, about their actual age, or older than their age. We also asked study members to rate their age perceptions in years: ‘How old do you feel?’ For informant impressions, informants who knew a study member well (94% response rate) were asked: ‘Compared to others their age, do you think they (the study member) look younger or older than others their age? Response options were: ‘much younger’, ‘a bit younger’, ‘about the same’, ‘a bit older’ or ‘much older’. For staff impressions, four members of the Dunedin Study unit staff completed a brief questionnaire describing each study member. To assess age appearance, staff used a seven-item scale to assign a ‘relative age’ to each study member (1 = young-looking, 7 = old-looking). Correlations between self-ratings, informant ratings and staff ratings ranged from 0.34 to 0.52. All reporters rated the study member’s general health using the following response options: excellent, very good, good, fair or poor. Correlations between self-ratings, informant ratings and staff ratings ranged from *r* = 0.48 to *r* = 0.55.

##### Cognitive functioning

The Wechsler Adult Intelligence Scale, Fourth Edition was administered at age 45 years, yielding the adult IQ. In addition to full-scale IQ, the Wechsler Adult Intelligence Scale, Fourth Edition yields indexes of four specific cognitive functional domains: processing speed; working memory; perceptual reasoning; and verbal comprehension. The Wechsler Intelligence Scale for Children-Revised was administered at ages 7, 9 and 11 years. To increase the baseline reliability, the three scores were averaged, yielding the childhood IQ. We measured cognitive decline by studying adult IQ scores after controlling for childhood IQ scores. We focused on change in overall IQ given evidence that aging-related slopes are correlated across all cognitive functions, indicating that research on cognitive decline may be best focused on a highly reliable summary index, rather than focused on individual functions^93^.

##### Facial age

Facial age was based on two measurements of perceived age by an independent panel of eight people. First, age range was assessed by an independent panel of four raters, who were presented with standardized (non-smiling) digital facial photographs of study members when they were 45 years old. Raters, who were kept blind to the actual age of study members, used a Likert scale to categorize each study member into a 5-year age range (that is, from 20 to 24 years old and up to 70+ years old). Interrater reliability was 0.77. The scores for each study member were averaged across all raters. Second, relative age was assessed by a different panel of four raters, who were told that all photos were of people aged 45 years old. These raters then used a 7-item Likert scale to assign a ‘relative age’ to each participant (that is, 1 = ‘young-looking’ to 7 = ‘old-looking’). Interrater reliability was 0.79. The measure of perceived age at 45 years (that is, facial age) was derived by standardizing and averaging age range and relative age scores.

#### HCP

The HCP is a publicly available dataset that includes 1,206 participants with extensive MRI data^49^. HCP data access is managed by the WU-Minn HCP consortium. All participants provided written informed consent. Specifically, we used data from 45 participants who completed the scan protocol a second time (with a mean interval between scans of approximately 140 days) allowing for the calculation of test–retest reliability. All participants were free of current psychiatric or neurological illness and were between 25 and 35 years of age. The mean age of the HCP test–retest sample analyzed was 30.3 years (s.d. = 3.3 years, range = 22–35 years) at the first time point.

##### MRI

Structural MRI data were analyzed using the HCP minimal preprocessing pipeline^94^. Briefly, T1-weighted images were processed using a custom FreeSurfer recon-all pipeline optimized for structural MRI with a higher resolution than 1 mm isotropic. Details on HCP MRI data acquisition have been described elsewhere^94^.

#### ADNI

The primary goal of ADNI is to test whether serial MRI, positron emission tomography, other biological markers and clinical and neuropsychological assessments can be combined to measure the progression of neurodegeneration in participants with MCI, AD and CN older adults (adni.loni.usc.edu)^95^. Cognitive and diagnostic data were downloaded on 12 June 2022. MRI data curated from the Alzheimer’s Disease Sequencing Project collection were downloaded on 7 December 2023. ADNI was approved by the institutional review boards of all the participating institutions. All participants provided written informed consent. The ADNI sample demographic information can be found in Supplementary Table 17.

##### MRI

T1-weighted scans were collected using either 1.5T or 3T scanners. MRI acquisition parameters varied across ADNI sites and waves; however, the targets for acquisition were isotropic 1-mm^3^ voxels^96^. Raw T1-weighted images were processed using longitudinal FreeSurfer v.6.0. Scans were excluded for low quality if they did not have a quality control rating of ‘pass’ from the ADNI investigators or if segmentation failed visual inspection. Scans were also excluded if participants were missing demographic data, such as age, sex or diagnosis (Supplementary Fig. 5). Further details on the MRI methods in ADNI can be found at adni.loni.usc.edu.

##### Cognitive and behavioral functioning

ADNI participants completed several cognitive and behavioral assessments at the time of scanning. The ADAS-Cog is a structured scale that evaluates memory, reasoning, language, orientation, ideational praxis and constructional praxis^97^. Delayed word recall and number cancellation are included in addition to the 11 standard ADAS items^98^. The test is scored for errors, ranging from 0 (best performance) to 85 (worst performance). The MMSE is a screening instrument that evaluates orientation, memory, attention, concentration, naming, repetition and comprehension, and ability to create a sentence and to copy two overlapping pentagons^99^. The MMSE is scored as the number of correctly completed items ranging from 0 (worst performance) to 30 (best performance). The MoCA is designed to detect people at the MCI stage of cognitive dysfunction^100^. The scale ranges from 0 (worst performance) to 30 (best performance). The RAVLT is a list learning task that assesses learning and memory. On each of five learning trials, 15 unrelated nouns are presented orally at the rate of one word per second and immediate free recall of the words is elicited. After a 30-min delay filled with unrelated testing, free recall of the original 15-word list is elicited. Both immediate recall and percentage forgotten are used. The LogMem tests I and II (delayed paragraph recall) are from the Wechsler Memory Scale-Revised. Free recall of one short story is elicited immediately after being read aloud to the participant and again after a 30-min delay. The total bits of information recalled after the delay interval (maximum score = 25) are analyzed. The Trail Making Test, Part B, consists of 25 circles, either numbered (one through 13) or containing letters (A through L). Participants connect the circles while alternating between numbers and letters (for example, A to 1, 1 to B, B to 2, 2 to C). Time to complete (300 s maximum) is the primary measure of interest. The FAQ is a self-report measure of instrumental ADLs, such as preparing meals, performing chores, keeping a schedule and traveling outside of one’s neighborhood^101^. Each unique cognitive testing measure was paired with the participant’s most temporally proximate brain scan within 6 months of cognitive testing.

##### Cognitive status

ADNI participants were classified into CN, MCI or dementia groups by ADNI study physicians based on subjective memory complaints, multiple neurocognitive and behavioral assessment scores, and level of impairment in ADLs. Complete diagnostic criteria can be found at adni.loni.usc.edu. Each individual scan was categorized according to the most temporally proximate cognitive diagnosis received by that participant.

##### Education

Education level was measured according to self-reported years of education. For the purposes of visualization in Fig. 5e, participants were grouped according to the following thresholds: less than high school: <12 years; high school: 12 years; some college: 12–15 years; college: 16 years; more than college: >16 years.

#### UKB

UKB is a UK population-based prospective study of 502,486 participants between the ages of 40 and 69 at baseline assessment^102^. We analyzed data from 42,583 participants who underwent brain MRI. The data used in these analyses were downloaded in April 2023. UKB was approved by the North West Multi-centre for Research Ethics Committee. All participants provided written informed consent. UKB sample demographic information can be found in Supplementary Table 17.

##### MRI

MRI methods for UKB have been described in detail elsewhere^103^. Briefly, MRI data were collected using three identical 3T Siemens Skyra scanners with a 32-channel Siemens head coil. T1-weighted images were obtained using a 3D MP-RAGE with the following parameters: TR = 2,000 ms; inversion time = 880 ms; 208 sagittal slices, matrix = 256 × 256 px; slice thickness = 1 mm with no gap; and total scan time = 4 min and 52 s. Our study made use of imaging-derived phenotypes generated using an image-processing pipeline developed and run on behalf of UKB^103^. As part of this pipeline, raw T1-weighted images were processed using the cross-sectional FreeSurfer v.6.0. All brain measures used in the cross-sectional analyses presented in this study were derived from the outputs of this FreeSurfer pipeline. We excluded UKB participants with a very low signal-to-noise ratio and highly unusual summary morphometrics indicative of low-quality reconstruction (Supplementary Fig. 6).

To measure change in hippocampal volume in the subset of UKB participants with longitudinal MRI data, we reprocessed all T1-weighted images for this subset of participants using the longitudinal FreeSurfer v.6.0 pipeline^104^. This allowed us to avoid the known biases that can be introduced by different processing stages of the longitudinal pipeline in different hardware and software environments. Specifically, we reprocessed both time points of each participant’s T1-weighted scans with the cross-sectional recon-all pipeline^105^. Then, we built an unbiased within-participant template^106^ using robust, inverse, consistent registration^107^ and reprocessed each T1-weighted scan through the automated longitudinal pipeline^104^.

##### Cognitive functioning

UKB participants completed a battery of cognitive tests at the time of MRI. We investigated cognitive functioning using the following measures: Reaction Time (field ID = 20023), Fluid Intelligence (field ID = 20016), Numeric Memory (field ID = 4282), Trail A (field ID = 6348) and Trail B (field ID = 6350), symbol digit substitution (field ID = 23324), Tower Rearranging (field ID = 21004) and Matrix Completion (field ID = 6373). The details of these cognitive tests have been described elsewhere^108^.

##### Frailty and self-reported health

To further investigate aging-related health, we used the Fried Frailty Index^53^. Briefly, the Fried Frailty Index is based on meeting the criteria for declining functioning across five domains: unintentional weight loss; exhaustion; weakness; physical inactivity; and slow walking speed. Index scores range from 0 to 5, with higher scores indicating greater frailty^109^. During their imaging visit, UKB participants were also asked to rate their overall health as ‘poor’, ‘fair’, ‘good’ or ‘excellent’. We used these ratings to investigate self-reported overall health (field ID = 2178).

##### Disease and mortality records

To assess the influence of DunedinPACNI on aging-related disease and mortality risk in UKB, we used variables from algorithmically defined health outcomes. Briefly, algorithmically defined outcomes are generated by combining information from baseline assessments (self-reported medical conditions, operations and medications) with linked data from hospital admissions and death registries. Because of the relatively small number of aging-related disease diagnoses at the follow-up, we defined aging-related morbidity as being diagnosed with myocardial infarction (field ID = 42000), chronic obstructive pulmonary disease (field ID = 42016), dementia (field ID = 42018) or stroke (field ID = 42006). Furthermore, we defined the risk of chronic disease as the emergence of one or more of these diagnoses among participants who were healthy at the time of scanning (that is, baseline). Mortality was quantified during the follow-up from death records (field ID = 40000).

##### Education, income and ethnicity

To test the association between DunedinPACNI and socioeconomic gradients of health, we tested whether UKB participants differed in DunedinPACNI scores as a function of educational attainment and household income. We grouped participants into three categories according to their self-reported educational qualifications (field ID = 6138) following prior work^110^. Specifically, these groups were: high (college or university degree); medium (A/AS level or equivalent or O level/GCSE or equivalent); and low (none of these). We also tested whether UKB participants differed in DunedinPACNI scores as a function of household income (field ID = 738).

We also conducted sensitivity analyses while restricting the UKB sample to either only low-income or only non-White participants. We considered participants as having a low income if they reported making less than £18,000 per year in household income. We considered participants to be non-White if they did not report their ethnic background (field ID = 21000) as ‘any other White background’, ‘British’, ‘do not know’, ‘Irish’, ‘prefer not to answer’ or ‘White’.

#### BrainLat

BrainLat is a multimodal neuroimaging dataset of patients with neurodegenerative diseases and healthy adult controls collected in Argentina, Chile, Colombia, Mexico and Peru^63^. We analyzed neuroimaging data from 368 individuals who were either cognitively healthy or diagnosed with AD or behavioral variant FTD. The BrainLat study was approved by the institutional ethical boards of each recruitment site. All participants, or their legal representatives, provided written informed consent. The BrainLat demographic data can be found in Supplementary Table 17.

##### MRI

The MRI methods for BrainLat have been described in detail elsewhere^63^. Briefly, T1-weighted MP-RAGE scans were collected on either 1.5 or 3T scanners. Acquisition parameters varied across sites, but scans most frequently had isometric 1-mm^3^ voxels. Scans were downloaded and then processed using FreeSurfer v.6.0. Participants were excluded if they failed the automated FreeSurfer quality metrics or a visual quality check of segmentation output (Supplementary Fig. 7).

##### Diagnostic classification

All participants included could speak fluent Spanish and had adequate visual and auditory capacity for testing. Participants were classified as CN if they had a modified clinical dementia rating of 0, an MMSE score above 25 and lacked a history of substance abuse, and neurological or psychiatric disorders. Patients were classified into the AD or FTD groups according to the National Institute of Neurological Disorders and Stroke–Alzheimer Disease and Related Disorders working group for probable AD or probable behavioral variant FTD. Diagnosis was supported using appropriate MRI or positron emission tomography imaging when needed^63^.

##### Cognitive status

BrainLat participants were evaluated with the MoCA. The MoCA is designed to detect people at the MCI stage of cognitive dysfunction^100^. The scale ranges from 0 (worst performance) to 30 (best performance).

### Statistical analyses

#### Pace of Aging

The derivation of Pace of Aging has been described elsewhere^1,2^. Briefly, we measured a panel of the following 19 biomarkers (Fig. 1a) at ages 26, 32, 38, and 45 years: BMI, waist/hip ratio, HbA1c, leptin, blood pressure (mean arterial pressure), cardiorespiratory fitness (VO_2_max), forced vital capacity ratio (FEV_1_/FVC), FEV_1_, total cholesterol, triglycerides, HDL, lipoprotein(a), apolipoprotein B100/A1 ratio, eGFR, blood urea nitrogen, hsCRP, white blood cell count, mean periodontal AL and the number of dental-caries-affected tooth surfaces (tooth decay). To calculate each study member’s Pace of Aging, we first transformed the biomarker values to a standardized scale. For each biomarker at each wave, we standardized values according to the age 26 distribution. Next, we calculated each study member’s slope for each of the 19 biomarkers using a mixed-effects growth model that regressed the biomarker’s level on age. Finally, we combined information from the 19 slopes of the biomarkers using a unit-weighting scheme. We calculated each study member’s Pace of Aging as the sum of age-dependent annual changes in biomarker *z*-scores. Biomarker standardization was performed separately for men and women.

#### DunedinPACNI

A schematic of DunedinPACNI model development can be found in Fig. 1. We trained an elastic net regression model to estimate the Pace of Aging from structural neuroimaging phenotypes in 860 Dunedin Study members at age 45 years (for the attrition analysis and inclusion criteria see Extended Data Figs. 1 and 2 and Supplementary Fig. 4). We selected 315 FreeSurfer measures as predictors from the following categories: regional CT, regional cortical SA, regional cortical GMV, regional cortical GWR and ‘ASEG’ volumes (that is, regional subcortical GMV, ventricular volumes and bilateral volume of white matter hypointensities). All cortical data were parcellated according to the Desikan–Killiany Atlas^111^. Note that although many ADNI scans do not pass quality control (Supplementary Fig. 5), FreeSurfer is a robust segmentation method, especially in healthy individuals^112^. Four phenotypes from the ‘ASEG’ volumes were excluded because of insufficient variance in the Dunedin Study (left and right white matter hypointensities, left and right non-white matter hypointensities). Model training was performed using the caret package in R. We conducted a grid search across a range of *α* and *λ* values. We used 100 repetitions of tenfold cross-validation to estimate model performance in held-out participants. The effect of sex was regressed from the Pace of Aging before model training. To prevent information leak during cross-validation, we regressed sex from each training set and applied the resulting *β* weights to each test set. This approach ensured that our model only used information from the training set, including covariate regression, when calculating predictions in each test set. We selected optimal tuning parameters according to the highest variance explained and lowest mean absolute error. The optimal tuning parameters were *α* = 0.214 and *λ* = 0.100. Using these parameters, we fitted the model to the entire *n* = 860 sample. The raw elastic net regression model weights can be found in Supplementary Table 18.

To generate DunedinPACNI scores in HCP, ADNI, UKB and BrainLat participants, we applied the regression weights from the DunedinPACNI model to FreeSurfer-derived phenotypes in each dataset and summed the products and model intercept. In ADNI, UKB and BrainLat, DunedinPACNI scores were correlated with chronological age (ADNI: *r* = 0.37; UKB: *r* = 0.50; BrainLat: *r* = 0.37; Supplementary Fig. 8).

In addition, we conducted the same procedure again without GWR because this measure is not always distributed in public datasets. We observed slightly reduced model accuracy when GWR was not included. DunedinPACNI estimates without GWR phenotypes showed excellent test–retest reliability in HCP. DunedinPACNI estimates were similar with and without GWR phenotypes in ADNI, UKB and BrainLat (see Supplementary Figs. 2 and 9 for more details).

#### Brain age gap

We submitted raw T1-weighted images from ADNI, UKB and BrainLat to the publicly available brainageR algorithm (v.2.1). This model, which has been described in detail elsewhere^113^, is trained to predict chronological age in a sample of healthy, cognitively unimpaired individuals aged 18–92 years. This algorithm was selected because it generates highly reliable estimates among published algorithms^64^. Briefly, brainageR is estimated by first segmenting and normalizing T1-weighted images using SPM12. Next, coefficients derived from a Gaussian process regression model predicting chronological age in a training dataset (*n* = 2,001) are applied to morphometric features from brain segmentations to predict participants’ chronological age. Brain age gap was subsequently estimated by subtracting actual chronological age from predicted age^113^. Notably, 15 ADNI scans failed the brain age gap pipeline (14 failed visual inspection of segmentation, one error when computing the predicted age). These scans were excluded from all brain age gap analyses, including comparative analyses with DunedinPACNI.

#### Dunedin Study validation analyses

To first test the validity of DunedinPACNI in the Dunedin Study training sample, we tested for linear associations between DunedinPACNI scores and one-legged balance, gait speed, step in place, chair stands, grip strength, visuomotor coordination, subjective physical limitations, subjective health, cognitive function, child-to-adult cognitive decline and facial aging while controlling for sex. We compared these effect sizes to associations between each of these measures and the original, longitudinal Pace of Aging.

#### Test–retest reliability

We used the HCP dataset to assess the test–retest reliability of DunedinPACNI. Reliability was quantified using a two-way mixed-effects intraclass correlation coefficient (3,1) with session modeled as a fixed effect, participant as a random effect and the test–retest interval as an effect of no interest^114^.

#### Cognitive and physical functioning

We first used linear regression models to test for associations between DunedinPACNI and scores on tests of cognition, physical function and health in ADNI and UKB. All analyses controlled for age and sex. In ADNI, we calculated robust standard errors to account for nonindependence from repeated observations. We also tested the standardized differences in DunedinPACNI scores between three groups based on cognitive status: CN, MCI and dementia. All group difference comparisons controlled for age and sex. We again calculated robust standard errors to account for nonindependence and conduced a sensitivity analysis while controlling for *APOE* ε4 carriership. We repeated these analyses with brain age gap. Notably, when conducting analyses on the combined effects of DunedinPACNI and brain age gap on cognitive outcomes in ADNI, we restricted the sample to the first time point of each measure. These analyses included only one observation per participant, allowing us to more easily combine effect sizes and CIs.

#### Dementia survival analysis

We conducted a Cox proportional hazards regression using the baseline DunedinPACNI scores of ADNI participants to predict their probability of cognitive decline or clinical conversion to dementia during the follow-up window. Conversion in CN participants was defined as having a diagnosis of CN at baseline but a diagnosis of MCI or dementia at the end of the follow-up. Conversion in participants with MCI was defined as having a diagnosis of MCI at baseline and a diagnosis of dementia by the end of the follow-up. Participants who had a baseline diagnosis of dementia or transitioned from MCI to CN were not included in this analysis. The analysis controlled for sex, age at baseline and length of the observation window. We investigated the influence of AD genetic risk on these results by conducting all analyses while additionally controlling for *APOE* ε4 carriership. We repeated these analyses for brain age gap.

#### Prediction of hippocampal atrophy rates

We used repeated MRI measurements from ADNI (*n* = 1,302) and UKB (*n* = 4,601) to generate estimates of change in hippocampal GMV. We used longitudinal ComBat on ADNI MRI data to remove differential scanner effects^115^. Next, using all available time points for each participant, we generated multilevel linear models for bilateral hippocampal volume with random effects for both participant and age. Using these models, we derived trajectories to track change in hippocampal GMV for each participant. We then tested whether each participant’s baseline DunedinPACNI scores could predict their subsequent rate of hippocampal atrophy. These analyses controlled for age, sex and length of the observation period. We investigated the influence of AD genetic risk on these results by conducting these analyses while additionally controlling for *APOE* ε4 carriership. We repeated these analyses for brain age gap.

#### Morbidity and mortality survival analyses

To investigate the association between DunedinPACNI and morbidity, we used UKB data to calculate the standardized differences in DunedinPACNI scores between three groups based on the number of lifetime chronic disease diagnosis (0, 1, 2+). Next, we conducted a Cox proportional hazards regression using UKB participants’ baseline DunedinPACNI scores to predict the onset of a chronic aging-related disease (*n* = 827 emergent diagnoses: myocardial infarction, chronic obstructive pulmonary disease, dementia or stroke) in participants who had never previously received any of these diagnoses at the time of scanning (*n* = 40,753). Similarly, to investigate the association between DunedinPACNI and mortality, we conducted a Cox proportional hazards regression using UKB participants’ baseline DunedinPACNI scores to predict death (*n* = 757 deaths). Both models controlled for baseline age, time to onset and sex. We repeated these analyses for brain age gap.

#### Socioeconomic inequality analyses

To investigate whether DunedinPACNI reflected gradients of socioeconomic inequality^57^, we first tested for linear relationships between DunedinPACNI and years of education in ADNI and UKB. We also tested for a linear relationship between DunedinPACNI and household income in UKB. These analyses controlled for sex and age. In ADNI, we included only the first MRI observation per participant.

#### Replication in a Latin American sample

To investigate whether DunedinPACNI generalizes to samples of individuals who are underrepresented in neuroimaging research^59^, we tested whether the degree of acceleration in DunedinPACNI among ADNI participants with dementia was similar in BrainLat participants with dementia. We tested for standardized differences by comparing the AD and FTD groups to the CN group, respectively. We also tested for linear associations between DunedinPACNI and MoCA scores. All analyses in this sample controlled for age and sex. We then compared the magnitude of acceleration among BrainLat participants with dementia to the previously identified acceleration among ADNI participants with dementia. Lastly, we compared the strength of the linear association between DunedinPACNI and MoCA scores in BrainLat participants to the previously identified association in ADNI participants.

#### Comparison with hippocampal and ventricular volume

We investigated how DunedinPACNI differs from two commonly used MRI-based measures of brain aging: hippocampal volume and ventricular volume. We calculated hippocampal volume as the sum of left and right hippocampal GMV measures derived from FreeSurfer. Likewise, we calculated ventricular volume as the sum of the left and right lateral ventricular volume measures. We first repeated cross-sectional associations with cognition, frailty and poor health in UKB, substituting DunedinPACNI with hippocampal volume or ventricular volume. Next, we conducted this same procedure with our Cox proportional hazards regression models of chronic disease and mortality risk in UKB, and cognitive decline risk among CN ADNI participants. Lastly, we compared coefficients for all analyses while including DunedinPACNI and either hippocampal volume or ventricular volume in the respective models. All analyses controlled for age and sex.

All visualizations were generated using the R package ggplot2 (ref. ^116^).

### Reporting summary

Further information on research design is available in the Nature Portfolio Reporting Summary linked to this article.

## Supplementary information

Supplementary Information Supplementary Figs. 1–9 and Tables 1–18.

Reporting Summary

Supplementary Table 18 Raw elastic net regression weights from the DunedinPACNI algorithm.

## Source data

Source Data Fig. 1 Statistical source data for Fig. 1.

Source Data Fig. 2 Statistical source data for Fig. 2.

Source Data Fig. 3 Statistical source data for Fig. 3.

Source Data Fig. 4 Statistical source data for Fig. 4.

Source Data Fig. 5 Statistical source data for Fig. 5.

Source Data Fig. 6 Statistical source data for Fig. 6.

Source Data Fig. 7 Statistical source data for Fig. 7.

Source Data Extended Data Fig. 1 Statistical source data for Extended Data Fig. 1.

Source Data Extended Data Fig. 2 Statistical source data for Extended Data Fig. 2.

Source Data Extended Data Fig. 3 Statistical source data for Extended Data Fig. 3.

Source Data Extended Data Fig. 5 Statistical source data for Extended Data Fig. 5.

Source Data Extended Data Fig. 6 Statistical source data for Extended Data Fig. 6.

Source Data Extended Data Fig. 7 Statistical source data for Extended Data Fig. 7.

Source Data Extended Data Fig. 8 Statistical source data for Extended Data Fig. 8.

Source Data Extended Data Fig. 9 Statistical source data for Extended Data Fig. 9.

## Figures and Tables

**Extended Data Fig. 4:**
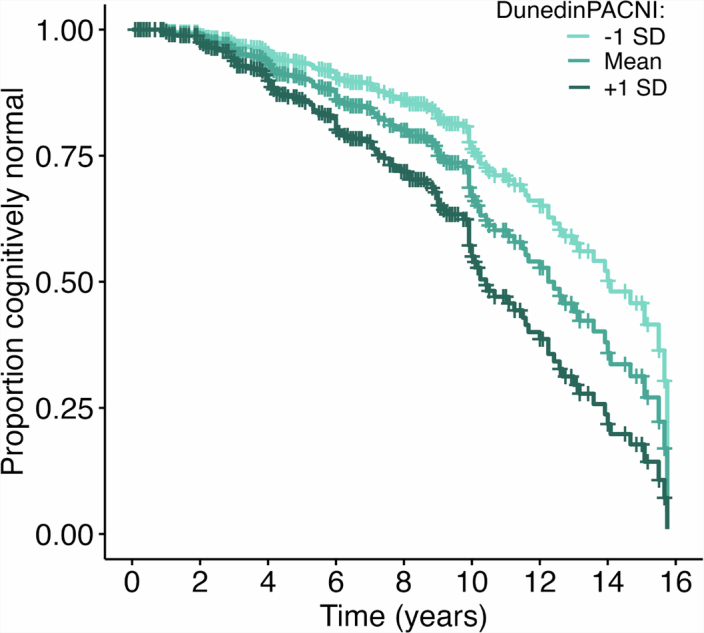
DunedinPACNI prediction of cognitive decline in ADNI with full follow-up window. Survival curve of the relative proportion of 624 cognitively normal ADNI participants at baseline who remained cognitively normal during the follow-up window grouped by slow, average, and fast baseline DunedinPACNI scores. Censored timepoints (that is, points when participants either converted to MCI/dementia or were lost to follow-up) are shown with cross marks. Note that relatively few participants have >9 years of follow-up. Abbreviations: ADNI = Alzheimer’s Disease Neuroimaging Initiative, SD = standard deviation.

## Data Availability

The Dunedin Study data are available via managed access. Researchers who wish to use the Dunedin Study data are invited to submit a concept paper proposing the data analysis project they wish to carry out, subject to the approval of the Dunedin Study investigators. Complete instructions on accessing the Dunedin Study data can be found at https://sites.duke.edu/moffittcaspiprojects/data-use-guidelines/. The HCP data are publicly available at www.humanconnectomeproject.org/data/. The ADNI data are publicly available at https://adni.loni.usc.edu/. Researchers can apply to access all UKB data at https://ams.ukbiobank.ac.uk/ams/. The BrainLat data are publicly available at www.synapse.org/Synapse:syn51549340/wiki/624187. Source data for Fig. 1b, Fig. 2a,b, Fig. 3a,b, Fig. 4b, Fig. 5a, Fig. 6 and Fig. 7, and Extended Data Figs. 1–3 and 5–9) are published with this article.

## Code availability

The DunedinPACNI algorithm is publicly available at GitHub (https://github.com/etw11/DunedinPACNI). All scripts used in the analyses presented in this article are publicly available at GitHub (https://github.com/etw11/WhitmanElliott_2024).
