# Supplementary material for: DunedinPACNI estimates the longitudinal Pace of Aging from a single brain image to track health and disease
Source: Nat Aging. 2025 Jul 1;5(8):1619–36. doi: 10.1038/s43587-025-00897-z (PMC12350157; doi:10.1038/s43587-025-00897-z)
Supplement: Supplementary file 1 — Supplementary Figs. 1–9 and Tables 1–18. [file 43587_2025_897_MOESM1_ESM.pdf]

nature aging

Supplementary information https://doi.org/10.1038/s43587-025-00897-z

DunedinPACNI estimates the longitudinal Pace of Aging from a single brain image to track health and disease

In the format provided by the authors and unedited

Table of Contents

Supplemental Figure S1. Visualization of elastic net feature importance scores..2
Supplemental Figure S2. DunedinPACNI has excellent test-retest reliability. 3
Supplemental Figure S3. DunedinPACNI is modestly correlated with brain age gap in ADNI, UK Biobank, and BrainLat...4
Supplemental Figure S4. Dunedin Study member inclusion flowchart. 5
Supplemental Figure S5. ADNI participant inclusion flowchart..6
Supplemental Figure S6. UK Biobank participant inclusion flowchart. 7
Supplemental Figure S7. BrainLat participant inclusion flowchart. 8
Supplemental Figure S8. DunedinPACNI is associated with age in ADNI, UK Biobank, and BrainLat participants..9
Supplemental Figure S9. DunedinPACNI is similar with and without gray-white signal intensity ratio phenotypes. 10
Supplemental Table S1. Associations between DunedinPACNI and cognition, physical function, and subjective aging in the Dunedin Study. 11
Supplemental Table S2. Associations between DunedinPACNI, brain age gap, and cognition in ADNI. .12
Supplemental Table S3. Associations between DunedinPACNI, brain age gap, and cognition in the UK Biobank..13
Supplemental Table S4. Associations with DunedinPACNI while excluding participants who go on to have cognitive decline or have high genetic risk for Alzheimer's Disease in the UK Biobank.................14
Supplemental Table S5. Associations between DunedinPACNI and hippocampal atrophy while controlling for APOE..15
Supplemental Table S6. Associations between DunedinPACNI, brain age gap and cognitive impairment in ADNI 16
Supplemental Table S7. Associations between DunedinPACNI, brain age gap and hippocampal atrophy17
Supplemental Table S8. Associations between DunedinPACNI, brain age gap and risk for cognitive decline, chronic disease, and death...18
Supplemental Table S9. Associations between DunedinPACNI, brain age gap and socioeconomic status 19
Supplemental Table S10. Associations between DunedinPACNI, brain age gap and dementia in the BrainLat sample...20
Supplemental Table S11. Associations between DunedinPACNI, brain age gap and cognitive status in the BrainLat sample...21
Supplemental Table S12. Associations between DunedinPACNI, hippocampal volume, and outcomes in UK Biobank participants. 22
Supplemental Table S13. Associations between DunedinPACNI, ventricular volume, and outcomes in UK Biobank participants. 23
Supplemental Table S14. Associations between DunedinPACNI, hippocampal volume, ventricular volume, and risk for cognitive decline, chronic disease, and death. 24
Supplemental Table S15. Associations between DunedinPACNI, brain age gap and outcomes in non-White UK Biobank participants...25
Supplemental Table S16. Associations between DunedinPACNI, brain age gap and outcomes in low-income UK Biobank participants...26
Supplemental Table S17. ADNI, UK Biobank, and Brainlat participant demographics 27

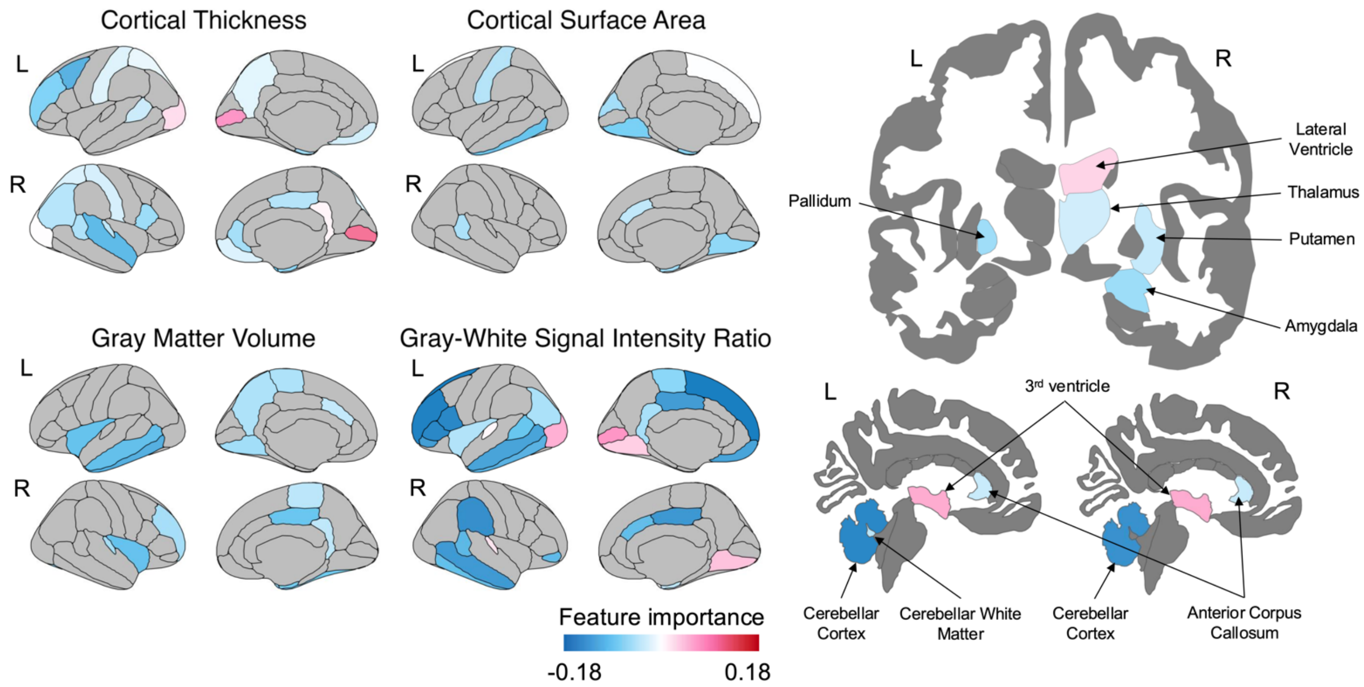

Supplemental Figure S1. Visualization of elastic net feature importance scores. Feature importance scores for the elastic net regression model predicting DunedinPACNI. Elastic net regression models systematically set highly correlated features equal to 0 in order to prevent overfitting. We chose elastic net regression due to the high correlations between brain features. Gray regions are set equal to 0 by the DunedinPACNI algorithm due to their correlations with other features. Thus, individual features inclusion/exclusion in the final DunedinPACNI model are not due to strong differences in feature importance of specific cortical regions or subcortical structures. We conducted a Haufe transformation to allow for feature interpretability. Warmer colors represent features that positively predicted DunedinPACNI scores (i.e., larger value indicates faster aging) while cooler colors represent features that negatively predicted DunedinPACNI scores (i.e., larger value indicates slower aging). Abbreviations: L = left, R = right.

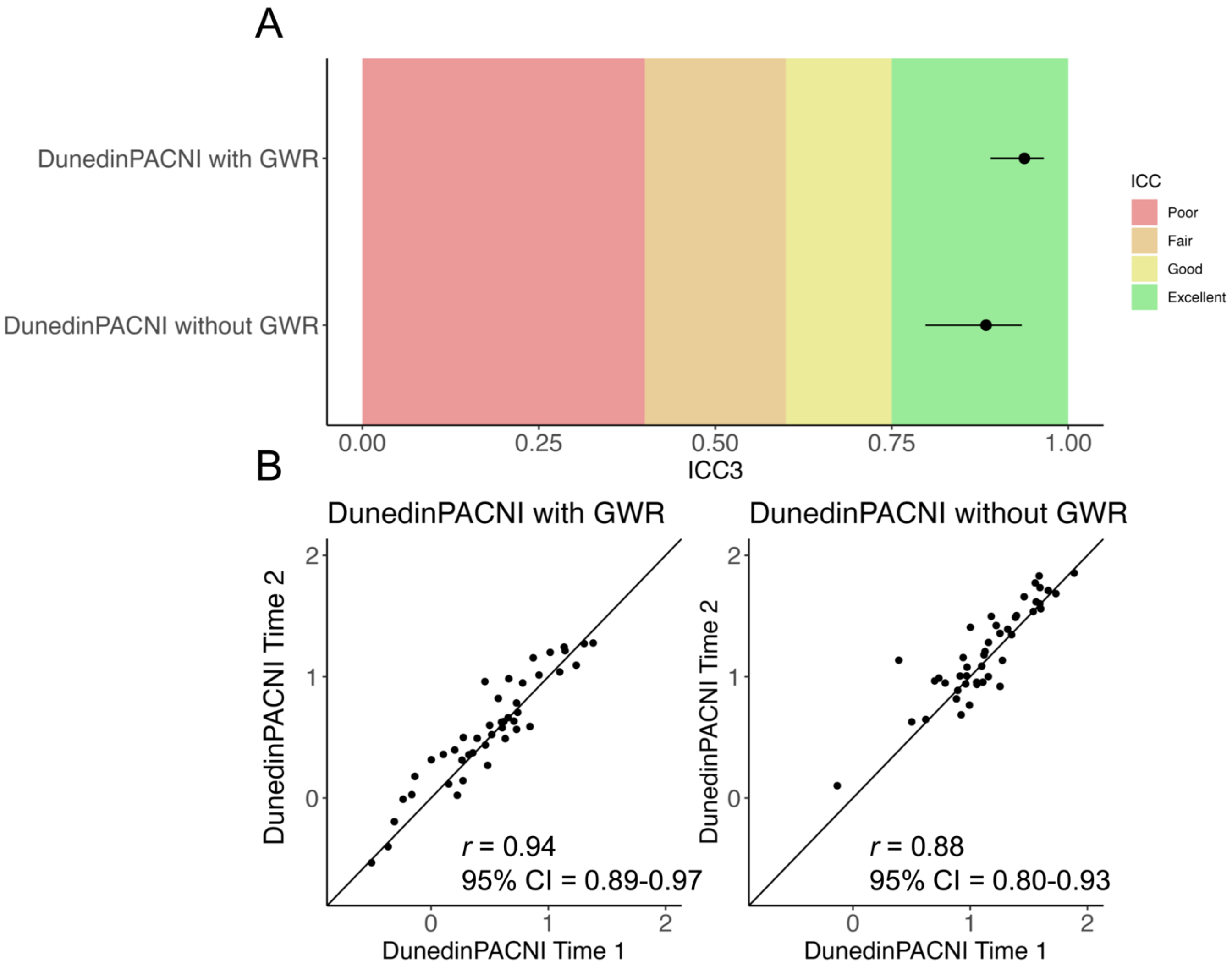

Supplemental Figure S2. DunedinPACNI has excellent test-retest reliability. A. Forest plot of intra-class correlations of DunedinPACNI scores calculated in the Human Connectome Project test-retest sample (N = 45). Intraclass correlation coefficients are plotted for DunedinPACNI both with and without gray-white signal intensity ratio measures. Error bars represent 95% confidence intervals. **B**. Correlation between DunedinPACNI estimates at scanning timepoints. Left panel shows DunedinPACNI without gray-white signal intensity ratio measures, right panel shows DunedinPACNI with gray-white signal intensity ratio measures. Both plots include an identity line. Abbreviations: CI = confidence interval, GWR = gray-white signal intensity ratio, ICC = intraclass correlation coefficient.

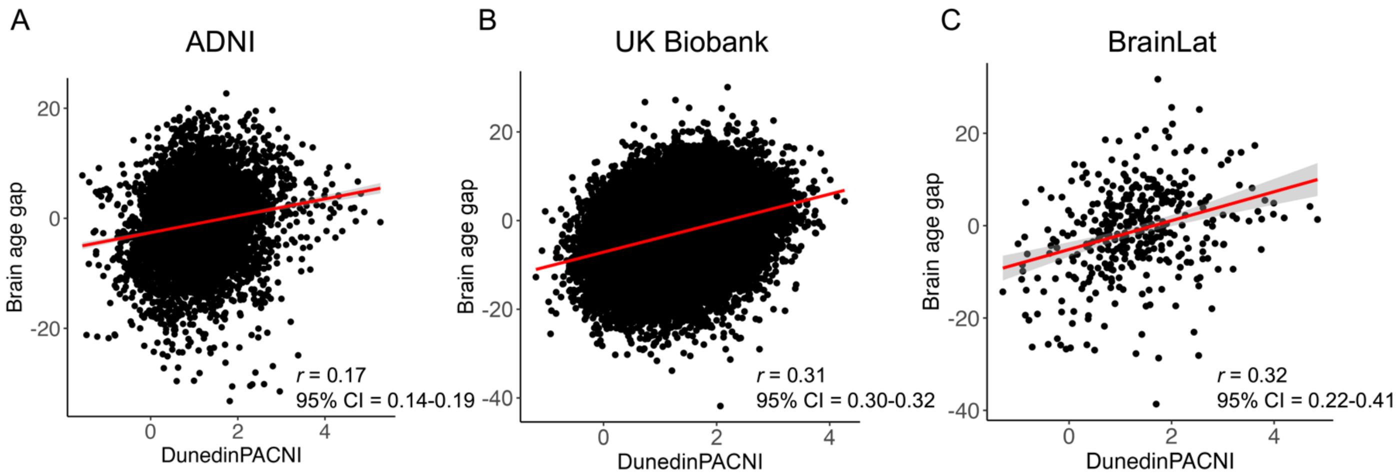

Supplemental Figure S3. DunedinPACNI is modestly correlated with brain age gap in ADNI, UK Biobank, and BrainLat. Correlation between DunedinPACNI and brain age gap in **A.** ADNI (N = 6,204 scans, N = 1,737 individuals), **B**. UK Biobank (N = 42,583), and **C.** BrainLat (N = 369). Error bands in all plots represent 95% confidence. ADNI = Alzheimer's Disease Neuroimaging Initiative, CI = confidence interval.

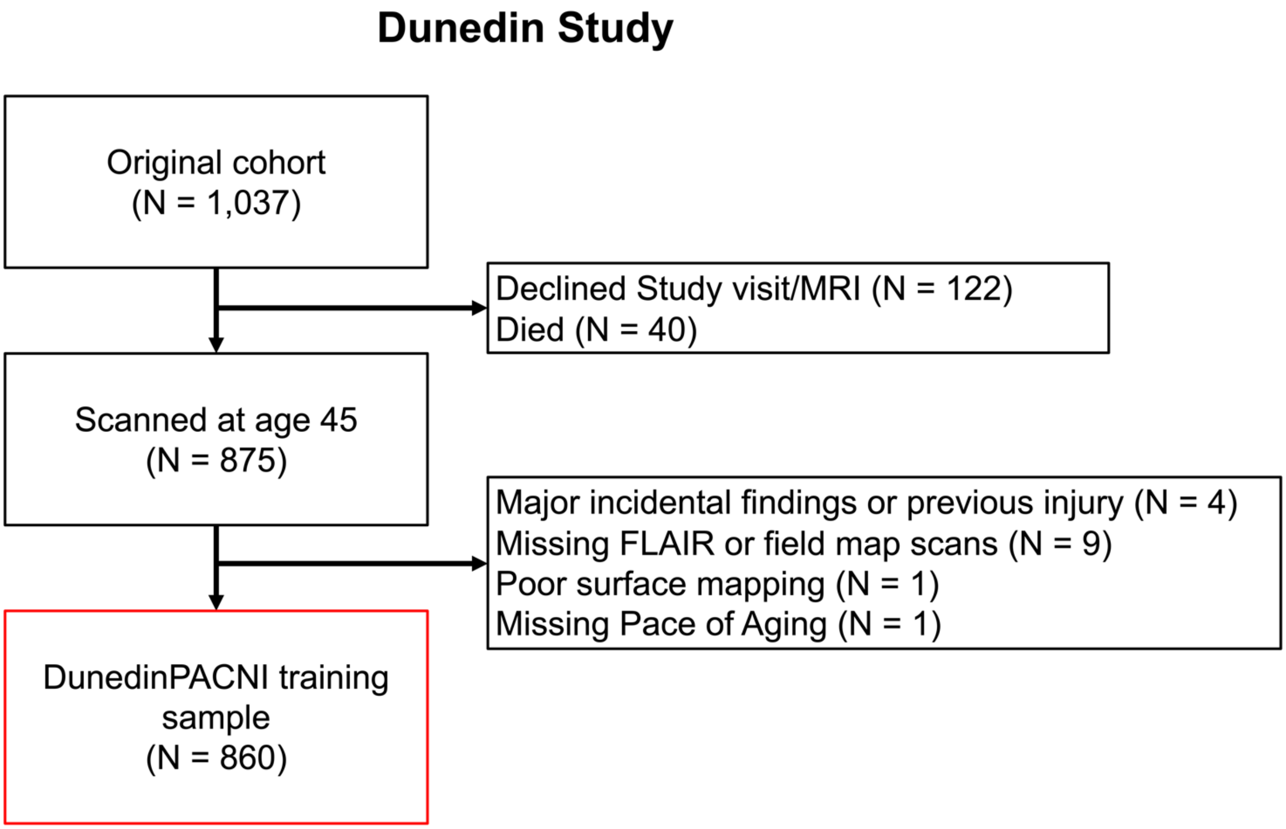

Supplemental Figure S4. Dunedin Study member inclusion flowchart. Abbreviations: FLAIR = Fluid attenuated inversion recovery, MRI = magnetic resonance imaging.

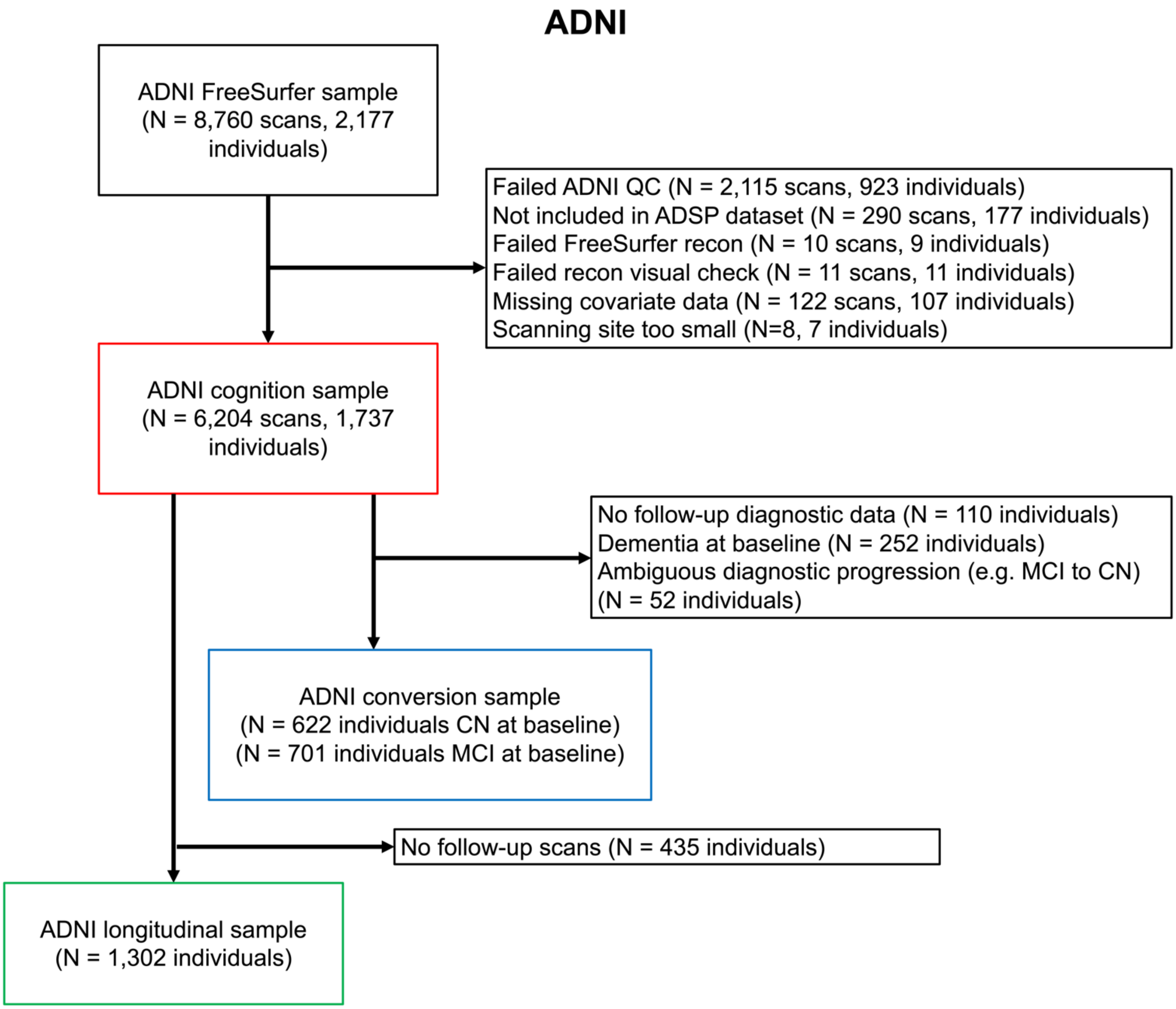

Supplemental Figure S5. ADNI participant inclusion flowchart. Participants were deemed to failed ADNI quality control if they were not given a "Pass" according to centralized quality control of FreeSurfer output by ADNI investigators or if their FreeSurfer output failed visual inspection. Abbreviations: ADNI = Alzheimer's Disease Neuroimaging Initiative, ADSP = Alzheimer's Disease Sequencing Project, CN = cognitively normal, MCI = mild cognitive impairment, QC = quality control.

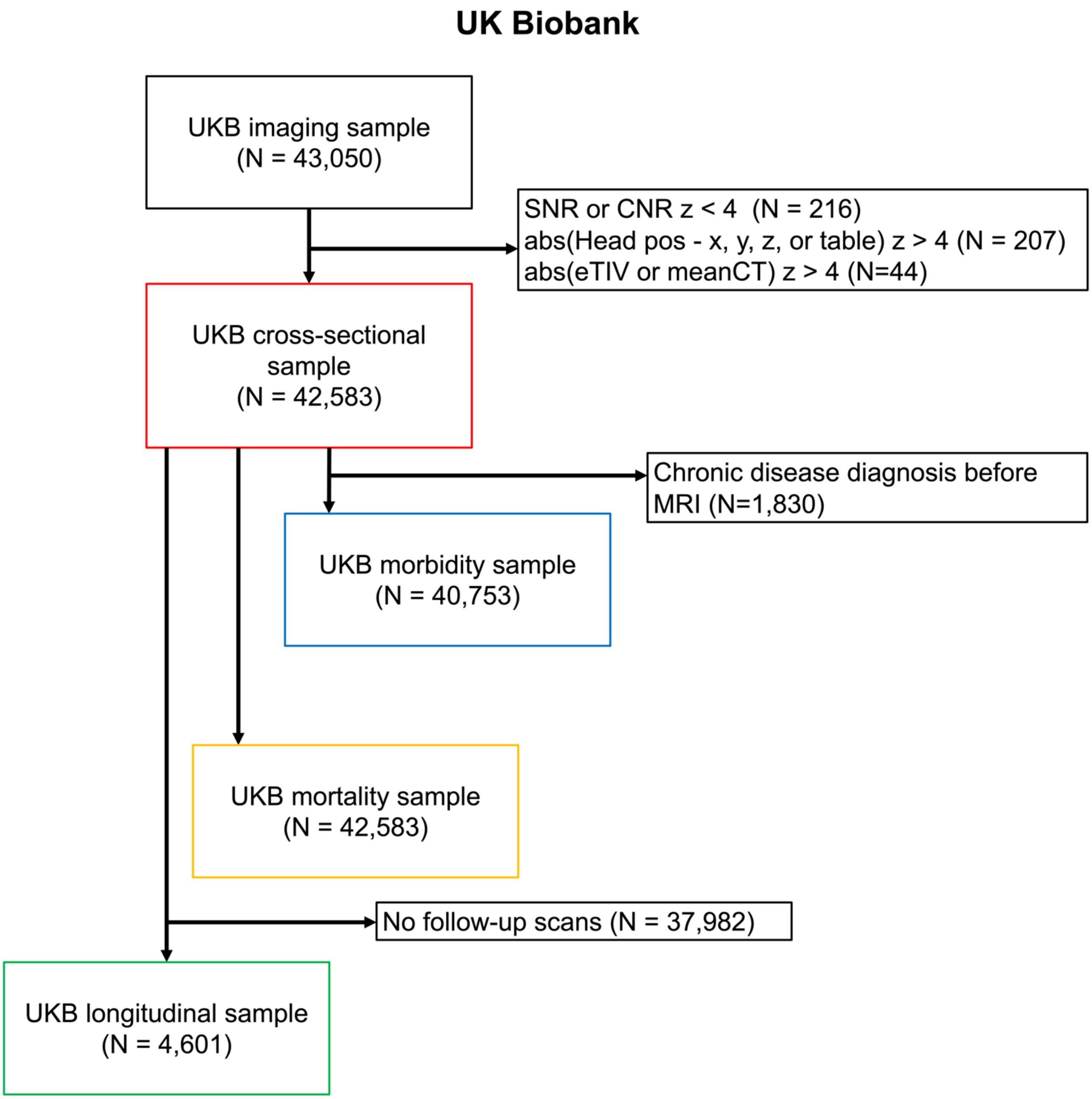

Supplemental Figure S6. UK Biobank participant inclusion flowchart. Participants were excluded for low scan signal-to-noise ratio, unusual head positioning, or extremely large intracranial volume or cortical thickness estimates presumed to represent low quality FreeSurfer reconstruction. Abbreviations: CNR = contrast-to-noise ratio, eTIV = estimated total intracranial volume, Head pos = head position, meanCT = mean cortical thickness, MRI = magnetic resonance imaging, SNR = signal-to-noise ratio, UKB = UK Biobank.

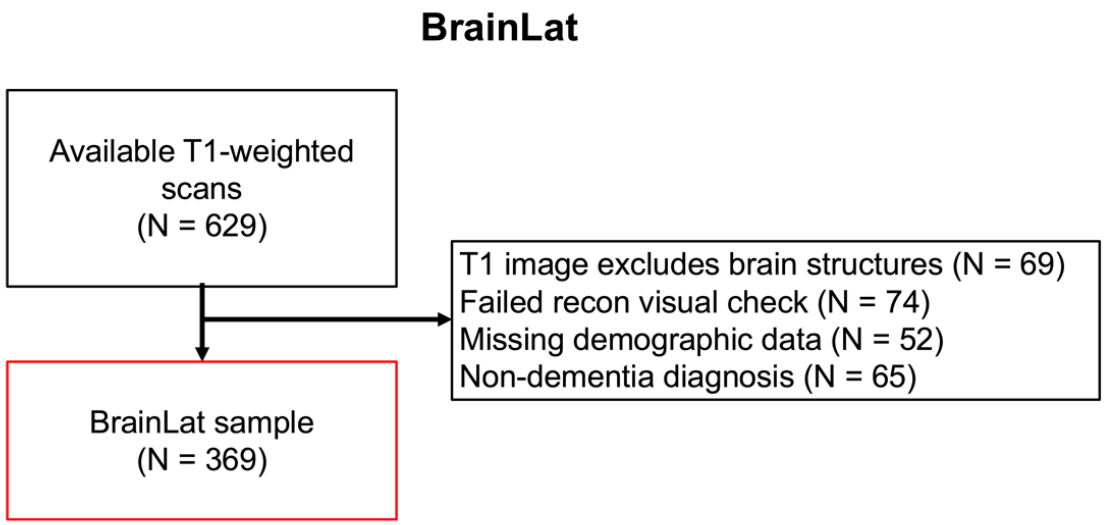

Supplemental Figure S7. BrainLat participant inclusion flowchart. Participants were excluded if their raw T1-weighted scans had incomplete whole-brain coverage or excessive motion corruption, missing demographic data, and diagnoses other than Alzheimer's dementia, frontotemporal dementia, or healthy control. Abbreviations: QC = quality control.

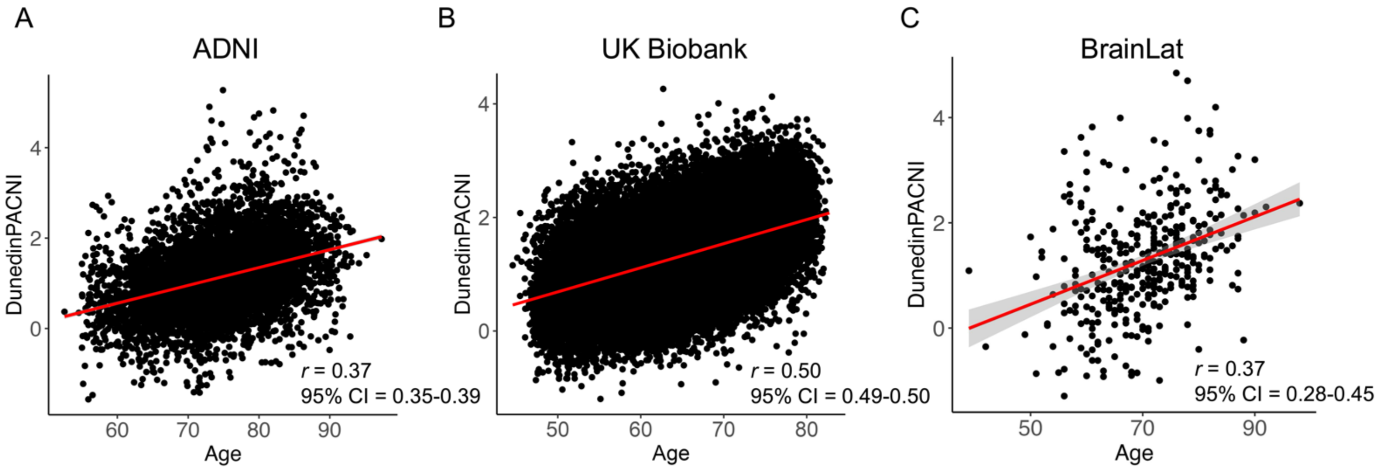

Supplemental Figure S8. DunedinPACNI is associated with age in ADNI, UK Biobank, and BrainLat participants. Correlation between age and DunedinPACNI in **A.** ADNI (N = 6,204 scans, N = 1,737 individuals), **B**. UK Biobank (N = 42,583), and **C.** BrainLat (N = 369). Error bands in all plots represent 95% confidence. ADNI = Alzheimer's Disease Neuroimaging Initiative, CI = confidence interval.

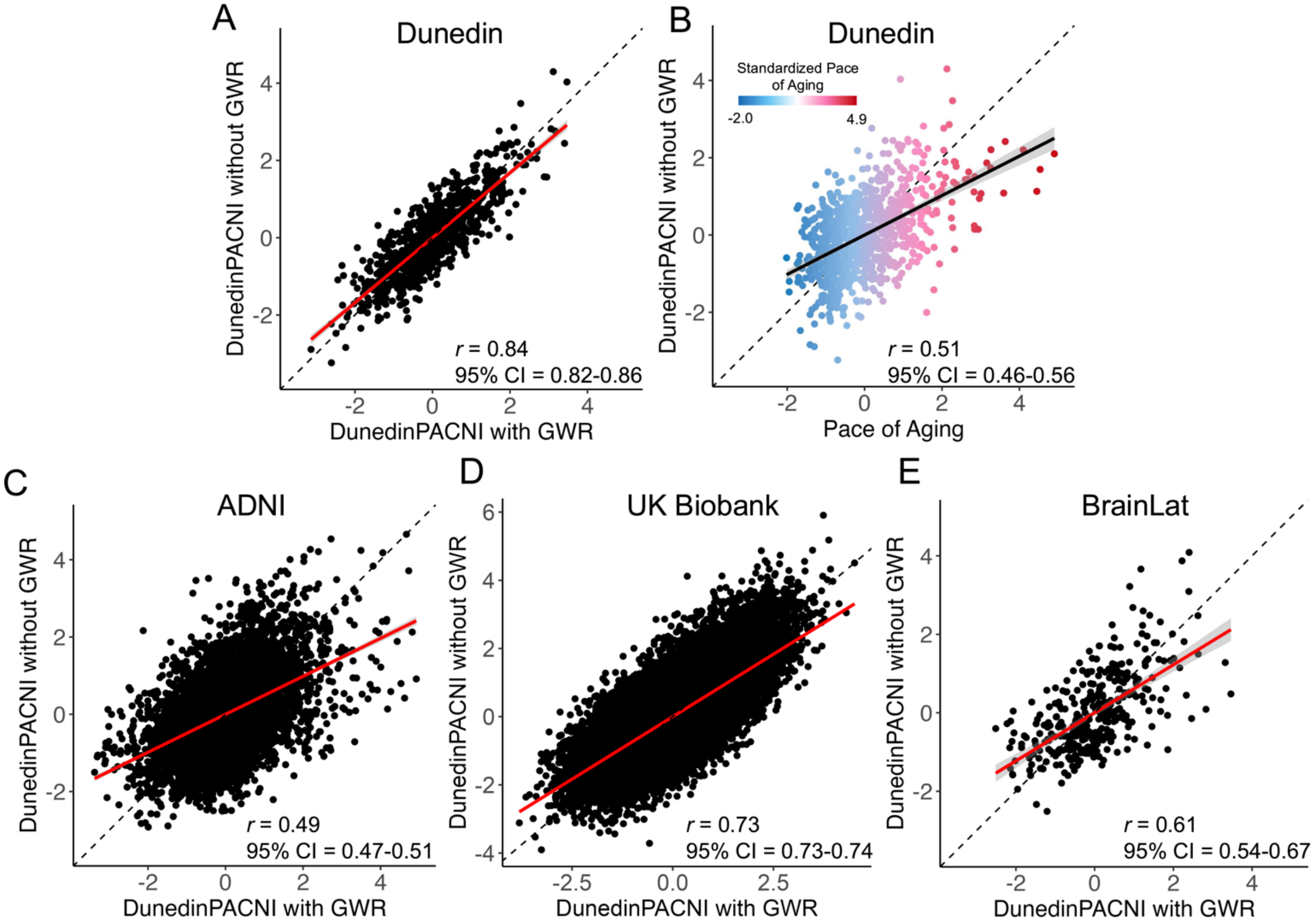

Supplemental Figure S9. DunedinPACNI is similar with and without gray-white signal intensity ratio phenotypes. DunedinPACNI can still be estimated when gray-white signal intensity ratio measures are not available. **A.** Correlation between DunedinPACNI estimated with and without gray-white signal intensity ratio measures in the Dunedin Study (N = 860). **B**. Correlation between the Pace of Aging and DunedinPACNI estimated without gray-white signal intensity ratio measures within the Dunedin Study (N = 860). Note that this correlation is likely higher than would be expected in an external dataset. Further note that Pace of Aging is standardized to mean = 0, SD = 1 to facilitate visualization. **C**. Correlation between DunedinPACNI estimated with and without gray-white signal intensity ratio in the ADNI cross-sectional sample (N = 6,204 scans, N = 1,737 individuals). **D.** Correlation between DunedinPACNI estimated with and without gray-white signal intensity ratio in the UK Biobank sample (N = 42,583). **E.** Correlation between DunedinPACNI estimated with and without gray-white signal intensity ratio in the BrainLat sample (N = 369). Error bands in all plots represent 95% confidence. Abbreviations: ADNI = Alzheimer's Disease Neuroimaging Initiative, CI = confidence interval, GWR = gray-white signal intensity ratio.

Supplemental Table S1. Associations between DunedinPACNI and cognition, physical function, and subjective aging in the Dunedin Study.

Phenotype	Measure	beta	p-value	95% CI lower bound	95% CI upper bound
**Balance**	DunedinPACNI	-0.26	<0.0001	-0.33	-0.19
	Pace of Aging	-0.36	<0.0001	-0.43	-0.30
**Gait speed**	DunedinPACNI	-0.26	<0.0001	-0.32	-0.19
	Pace of Aging	-0.32	<0.0001	-0.38	-0.26
**Step in place**	DunedinPACNI	-0.19	<0.0001	-0.26	-0.12
	Pace of Aging	-0.31	<0.0001	-0.37	-0.24
**Chair Stands**	DunedinPACNI	-0.23	<0.0001	-0.30	-0.17
	Pace of Aging	-0.31	<0.0001	-0.37	-0.24
**Grip strength**	DunedinPACNI	-0.36	<0.0001	-0.41	-0.31
	Pace of Aging	-0.39	<0.0001	-0.44	-0.34
**Motor coordination**	DunedinPACNI	0.24	<0.0001	0.17	0.30
	Pace of Aging	0.26	<0.0001	0.19	0.32
**Self-reported physical limitations**	DunedinPACNI	0.24	<0.0001	0.17	0.30
	Pace of Aging	0.28	<0.0001	0.21	0.34
**Self-rated health**	DunedinPACNI	-0.25	<0.0001	-0.31	-0.18
	Pace of Aging	-0.36	<0.0001	-0.42	-0.29
**Full-scale IQ**	DunedinPACNI	-0.27	<0.0001	-0.34	-0.21
	Pace of Aging	-0.31	<0.0001	-0.38	-0.25
**Verbal reasoning**	DunedinPACNI	-0.26	<0.0001	-0.33	-0.20
	Pace of Aging	-0.30	<0.0001	-0.36	-0.23
**Perceptual reasoning**	DunedinPACNI	-0.25	<0.0001	-0.31	-0.18
	Pace of Aging	-0.25	<0.0001	-0.32	-0.19
**Working memory**	DunedinPACNI	-0.17	<0.0001	-0.24	-0.11
	Pace of Aging	-0.20	<0.0001	-0.27	-0.14
**Processing speed**	DunedinPACNI	-0.15	<0.0001	-0.21	-0.08
	Pace of Aging	-0.22	<0.0001	-0.28	-0.15
**IQ decline**	DunedinPACNI	-0.12	0.0003	-0.19	-0.06
	Pace of Aging	-0.15	<0.0001	-0.22	-0.09
**Facial aging**	DunedinPACNI	0.26	<0.0001	0.19	0.32
	Pace of Aging	0.33	<0.0001	0.26	0.39

Abbreviations: CI = confidence interval, IQ = intelligence quotient.

Supplemental Table S2. Associations between DunedinPACNI, brain age gap, and cognition in ADNI.

		N			Independent models				Combined models			
**Cognitive measure**	**Brain measure**	**Observations**	**Individuals**	**Time difference**	**beta**	**p-value**	**95% CI lower bound**	**95% CI upper bound**	**beta**	**p-value**	**95% CI lower bound**	**95% CI upper bound**
ADAS-Cog	DunedinPACNI	5670	1721	3	0.39	< 0.001	0.34	0.43	0.33	< 0.001	0.28	0.37
ADAS-Cog	Brain age gap	5670	1721	3	0.31	< 0.001	0.27	0.36	0.24	< 0.001	0.19	0.28
MMSE	DunedinPACNI	5721	1725	3	-0.35	< 0.001	-0.40	-0.30	-0.31	< 0.001	-0.36	-0.26
MMSE	Brain age gap	5721	1725	3	-0.24	< 0.001	-0.29	-0.20	-0.17	< 0.001	-0.21	-0.13
MOCA	DunedinPACNI	3453	1206	4	-0.32	< 0.001	-0.38	-0.25	-0.28	< 0.001	-0.34	-0.21
MOCA	Brain age gap	3453	1206	4	-0.23	< 0.001	-0.30	-0.17	-0.19	< 0.001	-0.25	-0.13
RAVLT_mem	DunedinPACNI	5682	1719	3	-0.31	< 0.001	-0.35	-0.26	-0.25	< 0.001	-0.29	-0.21
RAVLT_mem	Brain age gap	5682	1719	3	-0.28	< 0.001	-0.32	-0.23	-0.22	< 0.001	-0.26	-0.17
RAVLT_learn	DunedinPACNI	5682	1719	3	-0.25	< 0.001	-0.28	-0.21	-0.21	< 0.001	-0.24	-0.17
RAVLT_learn	Brain age gap	5682	1719	3	-0.21	< 0.001	-0.25	-0.17	-0.16	< 0.001	-0.20	-0.12
RAVLT_forget	DunedinPACNI	5648	1715	3	0.18	< 0.001	0.14	0.22	0.15	< 0.001	0.11	0.19
RAVLT_forget	Brain age gap	5648	1715	3	0.16	< 0.001	0.12	0.21	0.13	< 0.001	0.09	0.17
LogMemory	DunedinPACNI	4616	1716	6	-0.32	< 0.001	-0.36	-0.28	-0.27	< 0.001	-0.31	-0.23
LogMemory	Brain age gap	4616	1716	6	-0.27	< 0.001	-0.32	-0.22	-0.21	< 0.001	-0.26	-0.16
DSST	DunedinPACNI	2193	622	2	-0.34	< 0.001	-0.41	-0.26	-0.31	< 0.001	-0.38	-0.23
DSST	Brain age gap	2193	622	2	-0.23	< 0.001	-0.32	-0.13	-0.16	0.001	-0.25	-0.07
TrailsB	DunedinPACNI	5559	1706	3	0.28	< 0.001	0.24	0.33	0.24	< 0.001	0.19	0.29
TrailsB	Brain age gap	5559	1706	3	0.22	< 0.001	0.17	0.26	0.16	< 0.001	0.12	0.21
FAQ	DunedinPACNI	5677	1715	3	0.35	< 0.001	0.30	0.40	0.31	< 0.001	0.26	0.35
FAQ	Brain age gap	5677	1715	3	0.24	< 0.001	0.19	0.29	0.17	< 0.001	0.12	0.21

Note: Time difference is in days.
Note: Combined models included both DunedinPACNI and brain age gap in the same model. Therefore results from the combined models show the effect for DunedinPACNI while controlling for brain age gap, and vice versa.
Abbreviations: ADAS = Alzheimer's Disease Assessment Scale – Cognitive Subscale, ADNI = Alzheimer's Disease Neuroimaging Initiative, CI = confidence interval, DSST = Digit Symbol Substitution Test, FAQ = Functional Assessment Questionnaire, LogMemory = Logical memory delayed recall, MMSE = Mini-Mental Status Exam, MOCA = Montreal Cognitive Assessment, RAVLT = Rey Auditory Verbal Learning Test, TrailsA = Trail Making Test Part A, TrailsB = Trail Making Test Part B.

Supplemental Table S3. Associations between DunedinPACNI, brain age gap, and cognition in the UK Biobank.

			Independent models				Combined models			
Cognitive measure	Brain measure	N	beta	p-value	95% CI lower bound	95% CI upper bound	beta	p-value	95% CI lower bound	95% CI upper bound
FluidIQ	DunedinPACNI	39164	-0.11	< 0.001	-0.12	-0.09	-0.10	< 0.001	-0.11	-0.09
FluidIQ	Brain age gap	39164	-0.04	< 0.001	-0.05	-0.03	-0.02	0.004	-0.03	-0.01
WM	DunedinPACNI	29696	-0.09	< 0.001	-0.10	-0.08	-0.09	< 0.001	-0.10	-0.07
WM	Brain age gap	29696	-0.03	< 0.001	-0.04	-0.01	-0.01	0.141	-0.02	0.00
VM	DunedinPACNI	28943	-0.09	< 0.001	-0.10	-0.08	-0.09	< 0.001	-0.10	-0.08
VM	Brain age gap	28943	-0.02	0.007	-0.03	0.00	0.00	0.802	-0.01	0.01
TrailsB	DunedinPACNI	28943	0.06	< 0.001	0.05	0.07	0.06	< 0.001	0.04	0.07
TrailsB	Brain age gap	28943	0.03	< 0.001	0.02	0.04	0.02	0.002	0.01	0.03
Matrix	DunedinPACNI	28626	-0.12	< 0.001	-0.13	-0.10	-0.11	< 0.001	-0.12	-0.10
Matrix	Brain age gap	28626	-0.04	< 0.001	-0.05	-0.03	-0.02	0.003	-0.03	-0.01
Tower	DunedinPACNI	28374	-0.05	< 0.001	-0.07	-0.04	-0.05	< 0.001	-0.07	-0.04
Tower	Brain age gap	28374	-0.02	0.003	-0.03	-0.01	-0.01	0.218	-0.02	0.00
DSST	DunedinPACNI	28645	-0.11	< 0.001	-0.12	-0.10	-0.10	< 0.001	-0.11	-0.09
DSST	Brain age gap	28645	-0.05	< 0.001	-0.06	-0.04	-0.03	< 0.001	-0.04	-0.02
TrailsA	DunedinPACNI	28943	0.06	< 0.001	0.04	0.07	0.05	< 0.001	0.04	0.06
TrailsA	Brain age gap	28943	0.03	< 0.001	0.02	0.04	0.02	0.002	0.01	0.03
RT	DunedinPACNI	39657	0.07	< 0.001	0.06	0.08	0.06	< 0.001	0.05	0.07
RT	Brain age gap	39657	0.03	< 0.001	0.03	0.04	0.02	< 0.001	0.01	0.03

Note: Combined models included both DunedinPACNI and brain age gap in the same model. Therefore results from the combined models show the effect for DunedinPACNI while controlling for brain age gap, and vice versa.
Abbreviations: CI = confidence interval, DSST = digit symbol substitution test, Matrix = Matrix Pattern Completion, IQ = intelligence quotient, RT = reaction time, TrailsA = Trail Making Test Part A, TrailsB = Trail Making Test Part B, Tower = Tower Rearranging, VM = visual memory, WM = working memory.

Supplemental Table S4. Associations with DunedinPACNI while excluding participants who go on to have cognitive decline or have high genetic risk for Alzheimer's Disease in the UK Biobank.

	Full sample					No double *APOE* E4 carriers or later dementia				
**Outcome**	**N**	**beta**	**p-value**	**95% CI lower bound**	**95% CI upper bound**	**N**	**beta**	**p-value**	**95% CI lower bound**	**95% CI upper bound**
FluidIQ	39164	-0.11	< 0.001	-0.12	-0.09	36236	-0.10	< 0.001	-0.12	-0.09
WM	29696	-0.09	< 0.001	-0.10	-0.08	27545	-0.09	< 0.001	-0.10	-0.07
VM	28943	-0.09	< 0.001	-0.10	-0.08	26863	-0.09	< 0.001	-0.10	-0.07
TrailsB	28943	0.06	< 0.001	0.05	0.07	26863	0.06	< 0.001	0.05	0.08
Matrix	28626	-0.12	< 0.001	-0.13	-0.10	26584	-0.12	< 0.001	-0.13	-0.10
Tower	28374	-0.05	< 0.001	-0.07	-0.04	26352	-0.05	< 0.001	-0.07	-0.04
DSST	28645	-0.11	< 0.001	-0.12	-0.10	26596	-0.11	< 0.001	-0.12	-0.09
TrailsA	28943	0.06	< 0.001	0.04	0.07	26863	0.06	< 0.001	0.04	0.07
RT	39657	0.07	< 0.001	0.06	0.08	36678	0.07	< 0.001	0.06	0.08
Frailty	42583	0.17	< 0.001	0.16	0.18	39337	0.16	< 0.001	0.14	0.17
Health	42235	-0.17	< 0.001	-0.18	-0.16	39029	-0.16	< 0.001	-0.17	-0.15

	Full sample					No double *APOE* E4 carriers or later dementia				
**Outcome**	**N**	**HR**	**p-value**	**95% CI lower bound**	**95% CI upper bound**	**N**	**HR**	**p-value**	**95% CI lower bound**	**95% CI upper bound**
Chronic disease	40753	1.14	0.001	1.05	1.23	37696	1.11	0.014	1.02	1.21
Mortality	42583	1.32	< 0.001	1.22	1.43	39337	1.29	< 0.001	1.18	1.40

Abbreviations: CI = confidence interval, DSST = Digit Symbol Substitution Task, HR = hazard ratio, IQ = intelligence quotient, Matrix = Matrix Pattern Completion, RT = Reaction Time, Tower = Tower Rearranging, TrailsA = Trail Making Test Part A, TrailsB = Trail Making Test Part B, VM = Visual Memory, WM = Working Memory.

Supplemental Table S5. Associations between DunedinPACNI and hippocampal atrophy while controlling for *APOE* E4 genotype

		Controlling for *APOE* E4 genotype				Original model			
Dataset	N	beta	p-value	95% CI lower bound	95% CI upper bound	beta	p-value	95% CI lower bound	95% CI upper bound
ADNI	1302	-0.14	< 0.001	-0.20	-0.08	-0.15	< 0.001	-0.21	-0.10
UK Biobank	4601	-0.09	< 0.001	-0.12	-0.06	-0.09	< 0.001	-0.12	-0.05

Abbreviations: ADNI = Alzheimer's Disease Neuroimaging Initiative, CI = confidence interval.

Supplemental Table S6. Associations between DunedinPACNI, brain age gap and cognitive impairment in ADNI

		N		Independent models				Combined models			
Diagnostic group	Brain measure	Individuals	Observations	beta	p-value	95% CI lower bound	95% CI upper bound	beta	p-value	95% CI lower bound	95% CI upper bound
MCI vs. CN	DunedinPACNI	1466	5064	0.27	< 0.001	0.18	0.35	0.23	< 0.001	0.14	0.31
MCI vs. CN	Brain age gap	1466	5064	0.24	< 0.001	0.14	0.34	0.19	< 0.001	0.09	0.29
Dementia vs. CN	DunedinPACNI	1201	3333	0.81	< 0.001	0.69	0.92	0.68	< 0.001	0.57	0.80
Dementia vs. CN	Brain age gap	1201	3333	0.68	< 0.001	0.57	0.80	0.52	< 0.001	0.40	0.64

Note: Combined models included both DunedinPACNI and brain age gap in the same model. Therefore results from the combined models show the effect for DunedinPACNI while controlling for brain age gap, and vice versa.
Abbreviations: ADNI = Alzheimer's Disease Neuroimaging Initiative, CI = confidence interval, CN = cognitively normal, MCI = mild cognitive impairment.

Supplemental Table S7. Associations between DunedinPACNI, brain age gap and hippocampal atrophy

			Indepdendent models				Combined models			
**Dataset**	**Brain model**	**N**	**beta**	**p-value**	**95% CI lower bound**	**95% CI upper bound**	**beta**	**p-value**	**95% CI lower bound**	**95% CI upper bound**
UK Biobank	DunedinPACNI	4601	-0.09	< 0.001	-0.12	-0.05	-0.09	< 0.001	-0.12	-0.06
UK Biobank	Brain age gap	4601	-0.01	0.646	-0.04	0.02	0.01	0.491	-0.02	0.04
ADNI	DunedinPACNI	1300	-0.15	< 0.001	-0.21	-0.10	-0.11	< 0.001	-0.17	-0.05
ADNI	Brain age gap	1300	-0.17	< 0.001	-0.23	-0.12	-0.14	< 0.001	-0.20	-0.09

Note: Combined models included both DunedinPACNI and brain age gap in the same model. Therefore results from the combined models show the effect for DunedinPACNI while controlling for brain age gap, and vice versa.
Abbreviations: ADNI = Alzheimer's Disease Neuroimaging Initiative, CI = confidence interval.

Supplemental Table S8. Associations between DunedinPACNI, brain age gap and risk for cognitive decline, chronic disease, and death

				Independent models				Combined models			
Outcome	Dataset	Brain variable	N	HR	p-value	95% CI lower bound	95% CI upper bound	HR	p-value	95% CI lower bound	95% CI upper bound
Cog. Decline (among CN)	ADNI	DunedinPACNI	622	1.49	0.005	1.12	1.97	1.40	0.023	1.05	1.86
Cog. Decline (among CN)	ADNI	Brain age gap	622	1.26	0.044	1.01	1.58	1.19	0.146	0.94	1.49
Dementia (among MCI)	ADNI	DunedinPACNI	701	1.44	< 0.001	1.26	1.65	1.37	< 0.001	1.19	1.56
Dementia (among MCI)	ADNI	Brain age gap	701	1.40	< 0.001	1.23	1.59	1.32	< 0.001	1.16	1.51
Chronic disease	UK Biobank	DunedinPACNI	40753	1.14	0.001	1.05	1.23	1.11	0.010	1.03	1.20
Chronic disease	UK Biobank	Brain age gap	40753	1.13	0.001	1.05	1.21	1.11	0.006	1.03	1.19
Mortality	UK Biobank	DunedinPACNI	42583	1.32	< 0.001	1.22	1.43	1.26	< 0.001	1.16	1.37
Mortality	UK Biobank	Brain age gap	42583	1.24	< 0.001	1.15	1.34	1.18	< 0.001	1.10	1.28

Note: Combined models included both DunedinPACNI and brain age gap in the same model. Therefore results from the combined models show the effect for DunedinPACNI while controlling for brain age gap, and vice versa.
Abbreviations: ADNI = Alzheimer's Disease Neuroimaging Initiative, CI = confidence interval, CN = cognitively normal, HR = hazard ratio, MCI = mild cognitive impairment.

Supplemental Table S9. Associations between DunedinPACNI, brain age gap and socioeconomic status

				Indepdendent models				Combined models			
Outcome	Dataset	Brain model	N	beta	p-value	95% CI lower bound	95% CI upper bound	beta	p-value	95% CI lower bound	95% CI upper bound
Years education	UK Biobank	DunedinPACNI	38297	-0.09	< 0.001	-0.10	-0.08	-0.10	< 0.001	-0.11	-0.08
Years education	UK Biobank	Brain age gap	38297	-0.01	0.053	-0.02	0.00	-0.01	0.053	-0.02	0.00
Years education	ADNI	DunedinPACNI	1734	-0.10	< 0.001	-0.15	-0.05	-0.10	< 0.001	-0.15	-0.05
Years education	ADNI	Brain age gap	1734	-0.01	0.608	-0.06	0.04	0.01	0.570	-0.04	0.06
Income	UK Biobank	DunedinPACNI	38297	-0.06	< 0.001	-0.07	-0.05	-0.06	< 0.001	-0.07	-0.05
Income	UK Biobank	Brain age gap	38297	-0.02	0.001	-0.03	-0.01	-0.02	0.001	-0.03	-0.01

Note: Combined models included both DunedinPACNI and brain age gap in the same model. Therefore results from the combined models show the effect for DunedinPACNI while controlling for brain age gap, and vice versa.
Abbreviations: ADNI = Alzheimer's Disease Neuroimaging Initiative, CI = confidence interval.

Supplemental Table S10. Associations between DunedinPACNI, brain age gap and dementia in the BrainLat sample

			Independent models				Combined models			
Diagnostic group	Brain variable	N	beta	p-value	95% CI lower bound	95% CI upper bound	beta	p-value	95% CI lower bound	95% CI upper bound
AD vs. CN	DunedinPACNI	285	0.70	< 0.001	0.49	0.91	0.38	< 0.001	0.17	0.58
FTD vs. CN	DunedinPACNI	207	0.79	< 0.001	0.55	1.04	0.51	< 0.001	0.28	0.75
AD vs. CN	Brain age gap	285	0.81	< 0.001	0.60	1.02	0.53	< 0.001	0.32	0.73
FTD vs. CN	Brain age gap	207	0.71	< 0.001	0.46	0.96	0.39	0.002	0.15	0.63

Note: Combined models included both DunedinPACNI and brain age gap in the same model. Therefore results from the combined models show the effect for DunedinPACNI while controlling for brain age gap, and vice versa.
Abbreviations: AD = Alzheimer's dementia, CI = confidence interval, CN = cognitively normal, FTD = frontotemporal dementia.

Supplemental Table S11. Associations between DunedinPACNI, brain age gap and cognitive status in the BrainLat sample

			Independent models				Combined models			
Measure	Brain variable	N	beta	p-value	95% CI lower bound	95% CI upper bound	beta	p-value	95% CI lower bound	95% CI upper bound
MoCA	DunedinPACNI	191	-0.35	< 0.001	-0.49	-0.20	-0.21	0.015	-0.38	-0.04
MoCA	Brain age gap	191	-0.33	< 0.001	-0.46	-0.20	-0.24	0.002	-0.39	-0.09

Note: Combined models included both DunedinPACNI and brain age gap in the same model. Therefore results from the combined models show the effect for DunedinPACNI while controlling for brain age gap, and vice versa.
Abbreviations: CI = confidence interval, MoCA = Montreal Cognitive Assessment.

Supplemental Table S12. Associations between DunedinPACNI, hippocampal volume, and outcomes in UK Biobank participants.

			Independent models				Combined models			
Outcome	Brain measure	N	beta	p-value	95% CI lower bound	95% CI upper bound	beta	p-value	95% CI lower bound	95% CI upper bound
FluidIQ	DunedinPACNI	39164	-0.11	< 0.001	-0.12	-0.09	-0.08	< 0.001	-0.09	-0.07
FluidIQ	Hippocampal volume	39164	-0.14	< 0.001	-0.15	-0.13	-0.13	< 0.001	-0.14	-0.12
WM	DunedinPACNI	29696	-0.09	< 0.001	-0.10	-0.08	-0.07	< 0.001	-0.09	-0.06
WM	Hippocampal volume	29696	-0.09	< 0.001	-0.11	-0.08	-0.08	< 0.001	-0.09	-0.07
VM	DunedinPACNI	28943	-0.09	< 0.001	-0.10	-0.08	-0.08	< 0.001	-0.10	-0.07
VM	Hippocampal volume	28943	-0.05	< 0.001	-0.06	-0.04	-0.03	< 0.001	-0.05	-0.02
TrailsB	DunedinPACNI	28943	0.06	< 0.001	0.05	0.07	0.05	< 0.001	0.04	0.06
TrailsB	Hippocampal volume	28943	0.05	< 0.001	0.04	0.07	0.04	< 0.001	0.03	0.06
Matrix	DunedinPACNI	28626	-0.12	< 0.001	-0.13	-0.10	-0.10	< 0.001	-0.11	-0.09
Matrix	Hippocampal volume	28626	-0.10	< 0.001	-0.12	-0.09	-0.09	< 0.001	-0.10	-0.07
Tower	DunedinPACNI	28374	-0.05	< 0.001	-0.07	-0.04	-0.04	< 0.001	-0.06	-0.03
Tower	Hippocampal volume	28374	-0.07	< 0.001	-0.08	-0.06	-0.06	< 0.001	-0.08	-0.05
DSST	DunedinPACNI	28645	-0.11	< 0.001	-0.12	-0.10	-0.10	< 0.001	-0.11	-0.08
DSST	Hippocampal volume	28645	-0.08	< 0.001	-0.10	-0.07	-0.07	< 0.001	-0.08	-0.05
TrailsA	DunedinPACNI	28943	0.06	< 0.001	0.04	0.07	0.05	< 0.001	0.03	0.06
TrailsA	Hippocampal volume	28943	0.06	< 0.001	0.05	0.08	0.05	< 0.001	0.04	0.07
RT	DunedinPACNI	39657	0.07	< 0.001	0.06	0.08	0.06	< 0.001	0.05	0.07
RT	Hippocampal volume	39657	0.05	< 0.001	0.04	0.06	0.04	< 0.001	0.03	0.05
Frailty	DunedinPACNI	42583	0.17	< 0.001	0.16	0.18	0.16	< 0.001	0.15	0.17
Frailty	Hippocampal volume	42583	0.07	< 0.001	0.06	0.08	0.04	< 0.001	0.03	0.05
Health	DunedinPACNI	42235	-0.17	< 0.001	-0.18	-0.16	-0.17	< 0.001	-0.18	-0.16
Health	Hippocampal volume	42235	-0.05	< 0.001	-0.06	-0.03	-0.01	0.008	-0.03	0.00

Abbreviations: CI = confidence interval, DSST = Digit Symbol Substitution Task, IQ = intelligence quotient, Matrix = Matrix Pattern Completion, RT = Reaction Time, Tower = Tower Rearranging, TrailsA = Trail Making Test Part A, TrailsB = Trail Making Test Part B, VM = Visual Memory, WM = Working Memory.

Supplemental Table S13. Associations between DunedinPACNI, ventricular volume, and outcomes in UK Biobank participants.

			Independent models				Combined models			
Outcome	Brain measure	N	beta	p-value	95% CI lower bound	95% CI upper bound	beta	p-value	95% CI lower bound	95% CI upper bound
FluidIQ	DunedinPACNI	39164	-0.11	< 0.001	-0.12	-0.09	-0.12	< 0.001	-0.13	-0.10
FluidIQ	Ventricular volume	39164	0.04	< 0.001	0.03	0.05	0.06	< 0.001	0.05	0.07
WM	DunedinPACNI	29696	-0.09	< 0.001	-0.10	-0.08	-0.09	< 0.001	-0.11	-0.08
WM	Ventricular volume	29696	0.01	0.236	-0.01	0.02	0.02	< 0.001	0.01	0.04
VM	DunedinPACNI	28943	-0.09	< 0.001	-0.10	-0.08	-0.09	< 0.001	-0.11	-0.08
VM	Ventricular volume	28943	0.00	0.455	-0.02	0.01	0.01	0.091	0.00	0.02
TrailsB	DunedinPACNI	28943	0.06	< 0.001	0.05	0.07	0.06	< 0.001	0.05	0.07
TrailsB	Ventricular volume	28943	0.02	0.006	0.01	0.03	0.01	0.247	-0.01	0.02
Matrix	DunedinPACNI	28626	-0.12	< 0.001	-0.13	-0.10	-0.12	< 0.001	-0.14	-0.11
Matrix	Ventricular volume	28626	0.02	0.006	0.01	0.03	0.04	< 0.001	0.03	0.05
Tower	DunedinPACNI	28374	-0.05	< 0.001	-0.07	-0.04	-0.05	< 0.001	-0.07	-0.04
Tower	Ventricular volume	28374	-0.01	0.337	-0.02	0.01	0.00	0.643	-0.01	0.02
TrailsA	DunedinPACNI	28943	0.06	< 0.001	0.04	0.07	0.05	< 0.001	0.04	0.07
TrailsA	Ventricular volume	28943	0.02	0.001	0.01	0.03	0.01	0.088	0.00	0.02
DSST	DunedinPACNI	28645	-0.11	< 0.001	-0.12	-0.10	-0.11	< 0.001	-0.12	-0.09
DSST	Ventricular volume	28645	-0.04	< 0.001	-0.05	-0.02	-0.02	0.004	-0.03	-0.01
RT	DunedinPACNI	39657	0.07	< 0.001	0.06	0.08	0.06	< 0.001	0.05	0.07
RT	Ventricular volume	39657	0.05	< 0.001	0.03	0.06	0.03	< 0.001	0.02	0.05
Frailty	DunedinPACNI	42583	0.17	< 0.001	0.16	0.18	0.17	< 0.001	0.16	0.18
Frailty	Ventricular volume	42583	0.04	< 0.001	0.03	0.05	0.01	0.031	0.00	0.02
Health	DunedinPACNI	42235	-0.17	< 0.001	-0.18	-0.16	-0.18	< 0.001	-0.19	-0.16
Health	Ventricular volume	42235	-0.02	0.001	-0.03	-0.01	0.01	0.012	0.00	0.03

Abbreviations: CI = confidence interval, DSST = Digit Symbol Substitution Task, IQ = intelligence quotient, Matrix = Matrix Pattern Completion, RT = Reaction Time, Tower = Tower Rearranging, TrailsA = Trail Making Test Part A, TrailsB = Trail Making Test Part B, VM = Visual Memory, WM = Working Memory.

Supplemental Table S14. Associations between DunedinPACNI, hippocampal volume, ventricular volume, and risk for cognitive decline, chronic disease, and death.

			Independent Models				Combined Models			
	Dataset	N	HR	p-value	95% CI lower bound	95% CI upper bound	HR	p-value	95% CI lower bound	95% CI upper bound
**Cog. Decline (among CN)**										
DunedinPACNI	ADNI	624	1.49	0.005	1.12	1.97	1.43	0.013	1.08	1.90
Hippocampal volume	ADNI	624	1.23	0.013	1.05	1.38	1.20	0.033	1.02	1.35
DunedinPACNI	ADNI	624	1.49	0.005	1.12	1.97	1.46	0.009	1.10	1.94
Ventricular volume	ADNI	624	1.12	0.208	0.94	1.33	1.07	0.481	0.89	1.27
**Chronic disease**										
DunedinPACNI	UK Biobank	40753	1.14	0.001	1.05	1.23	1.13	0.002	1.05	1.23
Hippocampal volume	UK Biobank	40753	1.06	0.117	0.98	1.15	1.04	0.330	0.96	1.13
DunedinPACNI	UK Biobank	40753	1.14	0.001	1.05	1.23	1.14	0.001	1.05	1.23
Ventricular volume	UK Biobank	40753	1.03	0.396	0.96	1.11	1.01	0.851	0.94	1.08
**Mortality**										
DunedinPACNI	UK Biobank	42583	1.32	< 0.001	1.22	1.43	1.30	< 0.001	1.20	1.41
Hippocampal volume	UK Biobank	42583	1.11	0.010	1.03	1.21	1.06	0.187	0.97	1.15
DunedinPACNI	UK Biobank	42583	1.32	< 0.001	1.22	1.43	1.27	< 0.001	1.17	1.38
Ventricular volume	UK Biobank	42583	1.18	< 0.001	1.11	1.26	1.13	< 0.001	1.06	1.20

Note: Hippocampal volume effect sizes directions are flipped to ease comparison with other measures.
Abbreviations: ADNI = Alzheimer's Disease Neuroimaging Initiative, CI = confidence interval, CN = cognitively normal, HR = hazard ratio

Supplemental Table S15. Associations between DunedinPACNI, brain age gap and outcomes in non-White UK Biobank participants.

	Non-White Sample					Full Sample				
Outcome measure	N	beta	p-value	95% CI lower bound	95% CI upper bound	N	beta	p-value	95% CI lower bound	95% CI upper bound
FluidIQ	1100	-0.06	0.074	-0.13	0.01	39164	-0.11	< 0.001	-0.12	-0.09
WM	855	-0.15	< 0.001	-0.23	-0.07	29696	-0.09	< 0.001	-0.10	-0.08
VM	834	-0.12	0.004	-0.20	-0.04	28943	-0.09	< 0.001	-0.10	-0.08
TrailsB	834	0.07	0.096	-0.01	0.15	28943	0.06	< 0.001	0.05	0.07
Matrix	812	-0.10	0.013	-0.18	-0.02	28626	-0.12	< 0.001	-0.13	-0.10
Tower	797	-0.04	0.400	-0.12	0.05	28374	-0.05	< 0.001	-0.07	-0.04
DSST	815	-0.10	0.008	-0.18	-0.03	28645	-0.11	< 0.001	-0.12	-0.10
TrailsA	834	0.14	0.001	0.06	0.22	28943	0.06	< 0.001	0.04	0.07
RT	1146	0.08	0.015	0.02	0.15	39657	0.07	< 0.001	0.06	0.08
Frailty	1289	0.11	0.001	0.05	0.18	42583	0.17	< 0.001	0.16	0.18
Health	1265	-0.21	< 0.001	-0.27	-0.15	42235	-0.17	< 0.001	-0.18	-0.16

Note: Analyses were not stratified by ancestry for hippocampal atrophy, disease risk, or mortality risk due to limited power.
Abbreviations: CI = confidence interval, DSST = Digit Symbol Substitution Task, IQ = intelligence quotient, Matrix = Matrix Pattern Completion, RT = Reaction Time, Tower = Tower Rearranging, TrailsA = Trail Making Test Part A, TrailsB = Trail Making Test Part B, VM = Visual Memory, WM = Working Memory.

Supplemental Table S16. Associations between DunedinPACNI, brain age gap and outcomes in low-income UK Biobank participants.

	Low-income Sample					Full Sample				
Outcome measure	N	beta	p-value	95% CI lower bound	95% CI upper bound	N	beta	p-value	95% CI lower bound	95% CI upper bound
FluidIQ	3882	-0.09	< 0.001	-0.13	-0.06	39164	-0.11	< 0.001	-0.12	-0.09
WM	2776	-0.12	< 0.001	-0.16	-0.08	29696	-0.09	< 0.001	-0.10	-0.08
VM	2703	-0.08	< 0.001	-0.12	-0.04	28943	-0.09	< 0.001	-0.10	-0.08
TrailsB	2703	0.09	< 0.001	0.04	0.13	28943	0.06	< 0.001	0.05	0.07
Matrix	2648	-0.12	< 0.001	-0.16	-0.07	28626	-0.12	< 0.001	-0.13	-0.10
Tower	2609	-0.06	0.004	-0.11	-0.02	28374	-0.05	< 0.001	-0.07	-0.04
DSST	2639	-0.14	< 0.001	-0.18	-0.10	28645	-0.11	< 0.001	-0.12	-0.10
TrailsA	2703	0.07	0.002	0.03	0.11	28943	0.06	< 0.001	0.04	0.07
RT	3990	0.08	< 0.001	0.04	0.11	39657	0.07	< 0.001	0.06	0.08
Frailty	4475	0.19	< 0.001	0.15	0.22	42583	0.17	< 0.001	0.16	0.18
Health	4412	-0.16	< 0.001	-0.19	-0.13	42235	-0.17	< 0.001	-0.18	-0.16

Note: Analyses were not stratified by income level for hippocampal atrophy, disease risk, or mortality risk due to limited power.
Abbreviations: CI = confidence interval, DSST = Digit Symbol Substitution Task, IQ = intelligence quotient, Matrix = Matrix Pattern Completion, RT = Reaction Time, Tower = Tower Rearranging, TrailsA = Trail Making Test Part A, TrailsB = Trail Making Test Part B, VM = Visual Memory, WM = Working Memory.

Supplemental Table S17. ADNI, UK Biobank, and Brainlat participant demographics

Dataset	N	Mean age (SD, range)	Sex (% male)
**ADNI**	1737	74.3 (7.2; 52.5-97.3)	48.3
cognitive decline (CN at baseline)	624	72.4 (6.3; 52.7-89.9)	57.2
conversion (MCI at baseline)	701	72.8 (7.3; 55.0-88.8)	43.4
longitudinal	1302	73.2 (7.1; 55.0-90.9)	48.8
**UK Biobank**	42583	64.4 (12.7; 44.6-82.8)	47.2
morbdity	40753	64.3 (12.7; 44.6-82.8)	46.2
mortality	42583	64.4 (12.7; 44.6-82.8)	47.2
longitudinal	4601	62.0 (11.6; 46.1-80.8)	47.3
**BrainLat**	369	70.2 (8.9; 39.0-98.0)	42.8
MoCA	191	68.7 (8.8; 39.0-98.0)	39.3

Abbreviations: ADNI = Alzheimer's Disease Neuroimaging Initative, CN = cognitively normal, MCI = mild cognitive impairment, MoCA = Montreal Cognitive Assessment, SD = standard deviation.
