# Supplementary material for: DunedinPACNI estimates the longitudinal Pace of Aging from a single brain image to track health and disease
Source: Nat Aging. 2025 Jul 1;5(8):1619–36. doi: 10.1038/s43587-025-00897-z (PMC12350157; doi:10.1038/s43587-025-00897-z)
Supplement: Supplementary file 2 — Reporting Summary [file 43587_2025_897_MOESM2_ESM.pdf]

nature portfolio

Corresponding author(s): Ethan Whitman

Last updated by author(s): 5/5/2025

Reporting Summary

Nature Portfolio wishes to improve the reproducibility of the work that we publish. This form provides structure for consistency and transparency in reporting. For further information on Nature Portfolio policies, see our Editorial Policies and the Editorial Policy Checklist.

Statistics

For all statistical analyses, confirm that the following items are present in the figure legend, table legend, main text, or Methods section.

n/a	Confirmed	
☐	☒	The exact sample size (n) for each experimental group/condition, given as a discrete number and unit of measurement
☐	☒	A statement on whether measurements were taken from distinct samples or whether the same sample was measured repeatedly
☐	☒	The statistical test(s) used AND whether they are one- or two-sided<br>*Only common tests should be described solely by name; describe more complex techniques in the Methods section.*
☐	☒	A description of all covariates tested
☐	☒	A description of any assumptions or corrections, such as tests of normality and adjustment for multiple comparisons
☐	☒	A full description of the statistical parameters including central tendency (e.g. means) or other basic estimates (e.g. regression coefficient) AND variation (e.g. standard deviation) or associated estimates of uncertainty (e.g. confidence intervals)
☐	☒	For null hypothesis testing, the test statistic (e.g. F, t, r) with confidence intervals, effect sizes, degrees of freedom and P value noted<br>*Give P values as exact values whenever suitable.*
☒	☐	For Bayesian analysis, information on the choice of priors and Markov chain Monte Carlo settings
☒	☐	For hierarchical and complex designs, identification of the appropriate level for tests and full reporting of outcomes
☐	☒	Estimates of effect sizes (e.g. Cohen's d, Pearson's r), indicating how they were calculated

Our web collection on statistics for biologists contains articles on many of the points above.

Software and code

Policy information about availability of computer code

Data collection: No software was used.

Data analysis: FreeSurfer v6.0 was used to process all neuroimaging data. DunedinPACNI was developed using the caret R package. Brain age gap was calculated using brainageR, which uses tools from SPM12. Visualizations were generated using the ggplot2 R package.

Custom code developed developed for this manuscript is available at https://github.com/etw11/WhitmanElliott_2024.

For manuscripts utilizing custom algorithms or software that are central to the research but not yet described in published literature, software must be made available to editors and reviewers. We strongly encourage code deposition in a community repository (e.g. GitHub). See the Nature Portfolio guidelines for submitting code & software for further information.

Data

Policy information about availability of data

All manuscripts must include a data availability statement. This statement should provide the following information, where applicable:

- Accession codes, unique identifiers, or web links for publicly available datasets
- A description of any restrictions on data availability
- For clinical datasets or third party data, please ensure that the statement adheres to our policy

Dunedin Study data is available via managed access at https://sites.duke.edu/moffittcaspiprojects/data-use-guidelines/. The Human Connectome Project data are

publicly available at http://www.humanconnectomeproject.org/data/. Alzheimer's Disease Neuroimaging Initiative data are publicly available at https://adni.loni.usc.edu/. Researchers can apply to access all UK Biobank data at https://ams.ukbiobank.ac.uk/ams/. BrainLat data is publicly available at https://www.synapse.org/Synapse:syn51549340/wiki/624187.

Research involving human participants, their data, or biological material

Policy information about studies with human participants or human data. See also policy information about sex, gender (identity/presentation), and sexual orientation and race, ethnicity and racism.

Reporting on sex and gender

We include biological sex as a covariate in all analyses and present the sex distribution of our samples in Methods section "Data Sources" and in Supplementary table S11. Data on gender was not included in this analysis.

Reporting on race, ethnicity, or other socially relevant groupings

Our research includes data from the United States, the United Kingdom, New Zealand, Argentina, Chile, Colombia, Mexico, and Peru. Categories of race, ethnicity, and socially relevant groupings differ across these countries. To retain this nuance, we have deferred to the categories and terms used by each study. In all cases, categories of race, ethnicity, and socially relevant groupings was self-reported by participants.

We tested the generalization of our findings to a Latin American sample by using BrainLat data. We did not analyze or report any data about the race and ethnicity of these participants, only their nationality.

In order to test the generalization of our analyses to non-White individuals in the UK, we performed a stratified analyses of UK Biobank participants who reported a 'non-White' ethnicity. The exact terminology and UK Biobank variable used to determine this grouping is found in the Methods in the 'UK Biobank' subsection.

Population characteristics

Dunedin Study:
N = 860, age = 45 years, 48% female.

HCP:
N = 45, mean age = 30.3 (SD = 3.3), 68.9% female.

ADNI:
N = 1,737 individuals, 6,204 scans, mean age = 74.3 (SD = 7.2), 51.7% female

UK Biobank:
N = 42,583, mean age = 64.4 (SD = 12.7), 52.8% female

BrainLat:
N = 369, mean age = 70.2 (SD = 8.9), 57.2% female

Demographic data for each ADNI, UK Biobank, and BrainLat subsample is summarized in Supplementary Table S17.

Recruitment

Dunedin Study:
Participants are members of the Dunedin Study, a longitudinal investigation of health and behavior in a representative birth cohort. All participants were born between April 1972 and March 1973 in Dunedin, New Zealand, were residents in the province and who participated in the first assessment at age 3 years.

HCP:
The HCP is a convenience sample of people free of psychiatric or neurologic illness between 25 and 35 years of age.

ADNI:
ADNI is a clinically recruited sample of memory clinic patients as well as cognitively normal older adult controls.

UK Biobank:
The UK Biobank is a UK population-based prospective study of adults between the ages of 40 and 69 at baseline assessment.

BrainLat:
The BrainLat study is a clinically recruited sample of patients with neurological disorders and healthy controls who participated in the Multi-Partner Consortium to Expand Dementia Research in Latin America (ReDLat). Participants were recruited from sites in Argentina, Chile, Colombia, Mexico, and Peru.

Ethics oversight

Dunedin Study:
The Dunedin Study was approved by the University of Otago Ethics Committee.

HCP:
The distribution of HCP data is overseen by the WU-Minn HCP consortium.

ADNI:
ADNI was approved by the Institutional Review Boards of all the participating institutions. The full list of ADNI sites can be found at adni.loni.usc.edu.

UK Biobank:
The UK Biobank was approved by the North West Centre for Research Ethics Committee.

BrainLat:

The BrainLat study was approved by the institutional ethics boards of each recruitment site. The full list of sites is listed in Prado et al., 2023, Scientific Data.

Note that full information on the approval of the study protocol must also be provided in the manuscript.

Field-specific reporting

Please select the one below that is the best fit for your research. If you are not sure, read the appropriate sections before making your selection.

☒ Life sciences ☐ Behavioural & social sciences ☐ Ecological, evolutionary & environmental sciences

For a reference copy of the document with all sections, see nature.com/documents/nr-reporting-summary-flat.pdf

Life sciences study design

All studies must disclose on these points even when the disclosure is negative.

Sample size

In all cases, we sought to obtain the largest available neuroimaging datasets of the populations in question. Because of this, we did not perform a prior power analyses.

Data exclusions

Dunedin Study:
We excluded participants for missing scans, missing data, low quality scans, or the presence of incidental findings or injury. Full details are presented in Supplemental Figure S4.

HCP:
We did not exclude any of the test-retest subjects.

ADNI:
We excluded participants who were missing data, failed QC, or were not included in the Alzheimer's Disease Sequencing Project image collection. Full details are presented in Supplemental Figure S5.

UK Biobank:
We excluded participants with low quality MRI data. Full details are presented in Supplemental Figure S6.

BrainLat:
We excluded participants with low quality MRI data, missing demographic data, and patients with neurological disorders other than dementia. Full details are presented in Supplemental Figure S7.

Replication

For all variables that are available in both ADNI and UK Biobank, or ADNI and BrainLat, all attempts at replication were successful. This includes a two cognitive tests (MoCA and Trail Making Test Part B), hippocampal atrophy, and educational attainment.

We were not able to replicate other findings due to unavailability of data.

Randomization

This is an observational study. As such, no randomization was performed.

Blinding

This is an observational study, As such, no blinding was performed.

Reporting for specific materials, systems and methods

We require information from authors about some types of materials, experimental systems and methods used in many studies. Here, indicate whether each material, system or method listed is relevant to your study. If you are not sure if a list item applies to your research, read the appropriate section before selecting a response.

Materials & experimental systems

n/a	Involved in the study	
☒	☐	Antibodies
☒	☐	Eukaryotic cell lines
☒	☐	Palaeontology and archaeology
☒	☐	Animals and other organisms
☒	☐	Clinical data
☒	☐	Dual use research of concern
☒	☐	Plants

Methods

n/a	Involved in the study	
☒	☐	ChIP-seq
☒	☐	Flow cytometry
☐	☒	MRI-based neuroimaging

Plants

Seed stocks	NA
Novel plant genotypes	NA
Authentication	NA

Magnetic resonance imaging

Experimental design

Design type

Structural MRI

Design specifications

There were no tasks or paradigms performed in the scanner for any Study.

Dunedin Study:
Study members were scanned one time as a part of their Phase 45 assessment.

HCP:
Subjects were scanned two times approximately 140 days apart.

ADNI:
Subjects were repeatedly scanned over variable intervals. Mean number of scans = 3.6 (SD = 2.2, min = 1, max = 13)

UK Biobank:
Subjects were scanned at the time of their study visit. A subset of participants (N = 4,601) were scanned a second time after approximately two years of follow up.

BrainLat:
Subjects were scanned at the time of their study visit. No longitudinal data was collected.

Behavioral performance measures

No task or paradigm was performed in the scanner.

Acquisition

Imaging type(s)

T1-weighted structural images.

Field strength

All Dunedin Study, HCP, and UK Biobank data was collected on 3 Tesla magnets. 4,138 ADNI scans were collected on 3 Tesla magnets and 2,066 ADNI scans were collected on 1.5 Tesla magnets. 194 BrainLat scans were collected on 3 Tesla magnets and 175 BrainLat scans were collected on 1.5 Tesla magnets.

Sequence & imaging parameters

Dunedin Study:
Dunedin Study members were scanned using a Siemens MAGNETOM Skyra (Siemens Healthcare GmbH) 3T scanner equipped with a 64-channel head/neck coil at the Pacific Radiology Group imaging center in Dunedin, New Zealand. High resolution T1-weighted images were obtained using an MP-RAGE sequence with the following parameters: TR=2400 ms; TE=1.98 ms; 208 sagittal slices; flip angle, 9°; FOV, 224 mm; matrix =256×256; slice thickness=0.9 mm with no gap (voxel size 0.9×0.875×0.875 mm); and total scan time=6 min and 52 s. 3D fluid-attenuated inversion recovery (FLAIR) images were obtained with the following parameters: TR=8000 ms; TE=399 ms; 160 sagittal slices; FOV=240 mm; matrix=232×256; slice thickness=1.2 mm (voxel size 0.9×0.9×1.2 mm); and total scan time=5 min and 38 s. Additionally, a gradient-echo field map was acquired with the following parameters: TR=712 ms; TE=4.92 and 7.38 ms; 72 axial slices; FOV=200 mm; matrix=100×100; slice thickness=2.0 mm (voxel size 2 mm isotropic); and total scan time=2 min and 25 s.

HCP:
HCP data were acquired using a custom Siemens scanner at Washington University in St. Louis using a standard 32-channel Siemens head coil and a "body" transmission coil designed by Siemens. T1-weighted images were acquired at -.7mm isotropic resolution. Full details of MRI acquisition in HCP are described in Van Essen et al., 2013, NeuroImage.

ADNI:
MRI acquisition parameters varied across ADNI sites and waves; however, the targets for acquisition were isotropic 1mm3 voxels. Further details on MRI acquisition in ADNI can be found at adni.loni.usc.edu.

UK Biobank:
MRI data in the UK Biobank were collected using 3 identical 3T Siemens Skyra scanners with a 32-channel Siemens head

coil. T1-weighted images were obtained using a 3D MP-RAGE with the following parameters: TR = 2000 ms; TI = 880 ms; 208 sagittal slices, matrix =256×256; slice thickness = 1 mm with no gap; and total scan time = 4 min and 52 s. Further details in MRI acquisition in UK Biobank are described in Alfaro-Almagro et al., 2018, NeuroImage.

BrainLat:
MRI acquisition parameters varied across BrainLat sites; however acquisition was generally isotropic 1mm3 voxels. Further details on MRI acquisition in BrainLat is described in Prado et al., 2023, Scientific Data. The full details of BrainLat MRI acquisition can be accessed at https://www.synapse.org/Synapse:syn51549340.

Area of acquisition: Whole brain

Diffusion MRI: ☐ Used ☒ Not used

Preprocessing

Preprocessing software:

Dunedin Study:
Structural MRI data were analyzed using the Human Connectome Project (HCP) minimal preprocessing pipeline. Briefly, T1-weighted and FLAIR images were processed through the PreFreeSurfer, FreeSurfer, and PostFreeSurfer pipelines. T1-weighted and FLAIR images were corrected for readout distortion using the gradient echo field map, coregistered, brain-extracted, and aligned together in the native T1 space using boundary-based registration. Images were then processed with a custom FreeSurfer recon-all pipeline that is optimized for structural MRI with a higher resolution than 1 mm isotropic.

HCP:
Structural MRI data was analyzed using FreeSurfer v6.0.

ADNI:
Structural MRI data was analyzed using FreeSurfer v6.0. Brain age gap scores were generated using brainageR (Biondo et al., 2022, NeuroImage: Clinical), which uses tools from SPM12.

UK Biobank:
Structural MRI data was analyzed using FreeSurfer v6.0. Brain age gap scores were generated using brainageR (Biondo et al., 2022, NeuroImage: Clinical), which uses tools from SPM12.

BrainLat:
Structural MRI data was analyzed using FreeSurfer v6.0. Brain age gap scores were generated using brainageR (Biondo et al., 2022, NeuroImage: Clinical), which uses tools from SPM12.

Normalization: Data were not normalized.

Normalization template: Data were not normalized to a common template.

Noise and artifact removal: We did not exclude participants for head motion.

Volume censoring: We did not use functional MRI data so we did not perform volume censoring.

Statistical modeling & inference

Model type and settings:

We generated an elastic net regression model based on FreeSurfer derived morphometrics in 860 people in the Dunedin Study. We also analyzed two summary brain variables: bilateral hippocampal volume and bilateral ventricle volume.

We did calculate the covariance between each MRI feature and our outcome variable as described in Haufe et al., 2014, NeuroImage to generate feature importance scores. This was for visualization purposes and we did not perform statistical tests on these scores.

Effect(s) tested:

We tested for linear relationships between cognition, frailty, health and hippocampal and ventricular volume, respectively. We also conducted Cox-proportional hazard regressions using hippocampal and ventricular volume respectively as predictors of time to cognitive decline, time to new chronic disease, and time to death.

Specify type of analysis: ☐ Whole brain ☐ ROI-based ☒ Both

Anatomical location(s): *Describe how anatomical locations were determined (e.g. specify whether automated labeling algorithms or probabilistic atlases were used).*

Statistic type for inference (See Eklund et al. 2016):

We conducted standard null hypothesis significance testing using whole brain measures (DunedinPACNI, brain age gap) and two a priori regional measures (hippocampal volume, ventricle volume).

Correction:

We did perform multiple testing correction due to the relatively low number of brain metrics being tested. To ward against false positive findings, we included multiple replication datasets and compared effect sizes across datasets.

Models & analysis

n/a	Involved in the study
☒	☐ Functional and/or effective connectivity
☒	☐ Graph analysis
☐	☒ Multivariate modeling or predictive analysis

Multivariate modeling and predictive analysis

We trained an elastic net regression model to estimate the Pace of Aging from structural neuroimaging phenotypes in 860 Dunedin Study members at age 45 (for attrition analysis and inclusion criteria see Supplemental Figures S1-S2, S7). We selected 315 variables as predictors from the following categories: regional cortical thickness (CT), regional cortical surface area (SA), regional cortical gray matter volume (GMV), regional cortical gray-white matter signal intensity ratio (GWR), and 'ASEG' volumes (i.e., regional subcortical gray matter volumes, ventricular volumes, and bilateral volume of white matter hypointensities). All cortical data were parcellated according to the Desikan-Killainy Atlas. Four phenotypes from the 'ASEG' volumes were excluded due to insufficient variance in the Dunedin Study (left/right white matter hypointensities, left/right non-white matter hypointensities). Model training was performed using the caret package in R. We conducted a grid search across a range of alpha and lambda values. We used 100 repetitions of 10-fold cross-validation to estimate model performance in held-out participants. The effect of sex was regressed from the Pace of Aging prior to model training. To prevent information leak during cross-validation, we regressed sex from each training set and applied the resulting beta weights to each test set. This approach ensured that our model only used information from the training set, including covariate regression, when calculating predictions in each test set. We selected optimal tuning parameters according to highest variance explained and lowest mean absolute error. The optimal tuning parameters were alpha = 0.214 and lambda = 0.100. Using these parameters, we fit the model to the entire N=860 sample.

To generate DunedinPACNI scores in HCP, ADNI, UK Biobank, and BrainLat participants, we applied the regression weights from the DunedinPACNI model to FreeSurfer-derived phenotypes within each dataset and summed the products and model intercept.

Brain age gap scores in ADNI, UK Biobank, and BrainLat were generated using the brainageR software package found at https://github.com/james-cole/brainageR .
